# Multiplexed non-invasive tumor imaging of glucose metabolism and receptor-ligand engagement using dark quencher FRET acceptor

**DOI:** 10.7150/thno.45825

**Published:** 2020-08-15

**Authors:** Alena Rudkouskaya, Nattawut Sinsuebphon, Marien Ochoa, Sez-Jade Chen, Joseph E. Mazurkiewicz, Xavier Intes, Margarida Barroso

**Affiliations:** 1 Department of Cellular and Molecular Physiology, Albany Medical College, Albany, NY 12208, USA.; 2 Center for Modeling, Simulation, and Imaging in Medicine, Rensselaer Polytechnic Institute, Troy, NY 12180, USA.; 3 Department of Neuroscience and Experimental Therapeutics, Albany Medical College, Albany, NY 12208, USA.

**Keywords:** target engagement, metabolism, breast cancer, lifetime imaging, FRET

## Abstract

**Rationale:** Following an ever-increased focus on personalized medicine, there is a continuing need to develop preclinical molecular imaging modalities to guide the development and optimization of targeted therapies. Near-Infrared (NIR) Macroscopic Fluorescence Lifetime Förster Resonance Energy Transfer (MFLI-FRET) imaging offers a unique method to robustly quantify receptor-ligand engagement in live intact animals, which is critical to assess the delivery efficacy of therapeutics. However, to date, non-invasive imaging approaches that can simultaneously measure cellular drug delivery efficacy and metabolic response are lacking. A major challenge for the implementation of concurrent optical and MFLI-FRET *in vivo* whole-body preclinical imaging is the spectral crowding and cross-contamination between fluorescent probes.

**Methods:** We report on a strategy that relies on a dark quencher enabling simultaneous assessment of receptor-ligand engagement and tumor metabolism in intact live mice. Several optical imaging approaches, such as *in vitro* NIR FLI microscopy (FLIM) and *in vivo* wide-field MFLI, were used to validate a novel donor-dark quencher FRET pair. IRDye 800CW 2-deoxyglucose (2-DG) imaging was multiplexed with MFLI-FRET of NIR-labeled transferrin FRET pair (Tf-AF700/Tf-QC-1) to monitor tumor metabolism and probe uptake in breast tumor xenografts in intact live nude mice. Immunohistochemistry was used to validate *in vivo* imaging results.

**Results:** First, we establish that IRDye QC-1 (QC-1) is an effective NIR dark acceptor for the FRET-induced quenching of donor Alexa Fluor 700 (AF700). Second, we report on simultaneous *in vivo* imaging of the metabolic probe 2-DG and MFLI-FRET imaging of Tf-AF700/Tf-QC-1 uptake in tumors. Such multiplexed imaging revealed an inverse relationship between 2-DG uptake and Tf intracellular delivery, suggesting that 2-DG signal may predict the efficacy of intracellular targeted delivery.

**Conclusions:** Overall, our methodology enables for the first time simultaneous non-invasive monitoring of intracellular drug delivery and metabolic response in preclinical studies.

## Introduction

Skyrocketing costs of new targeted drug development, combined with a high failure rate of clinical trials, calls for a new paradigm of drug delivery assessment in living intact small animals. Especially, it is critical to characterize the extent and duration of drug-target engagement in an undisturbed tumor environment to determine the clinical efficacy of new anti-cancer drugs in preclinical studies 1-3. However, a major challenge in preclinical molecular imaging is the lack of multiplexing approaches that can directly assess intracellular anti-cancer drug delivery while also reporting on drug efficacy via tumor metabolic signatures in live animals, non-invasively and longitudinally 4-7. Indeed, current standard approaches are either destructive or incapable of discriminating between tumor cell-associated drugs and unbound drugs residing in the extracellular space, and simultaneously monitoring tumor metabolic levels 8-11. Currently, drug target engagement and efficacy assessment data are typically collected from different animals, increasing the complexity of preclinical studies, due to extensive inter-tumor heterogeneity.

Monitoring drug biodistribution and quantification in preclinical *in vivo* studies greatly benefits from different advanced imaging methods, such as Fluorescence Lifetime Imaging (FLI). In particular, FLI has been used to measure Förster Resonance Energy Transfer (FRET) events to examine protein-protein interactions in cells and organs, *in vitro* and *in vivo*. FRET can occur when a donor fluorophore is in nanometer-range proximity (2-10 nm) of an acceptor molecule. Upon donor excitation, FLI estimates the occurrence of FRET, by determining the reduction of the fluorescence lifetime of the donor molecule 12-14. When applied to receptor-ligand systems, FRET occurs when donor-labeled and acceptor-labeled ligands/antibodies bind to dimerized or cross-linked receptors 15-21. Hence, FLI FRET acts as a direct reporter of receptor engagement and internalization via the measurement of the fraction of donor-labeled entity undergoing binding to its respective receptor and subsequent internalization.

Recently, Near-Infrared (NIR) Macroscopic FLI-FRET (MFLI-FRET) has been developed to quantitatively report on the intracellular delivery of drug-protein carriers, providing an online readout of the true payload delivered to tumor cells in live intact animals 22-25. However, to extend the utility of this methodology to the non-invasive monitoring of tumor metabolism and receptor-ligand target engagement, it is crucial to enable concurrent imaging of NIR fluorescent metabolic probes and NIR FLI probes in the same tumor xenograft in a live intact animal.

Bioenergetic reprogramming of cancer cells to aerobic glycolysis is a hallmark of neoplastic diseases 26. Metabolic imaging is an important tool to evaluate drug response in tumors by monitoring the reduction in glycolytic activity in preclinical and clinical oncology research 27. Although ^18^F-fluorodeoxyglucose (FDG) is the standard functional readout of glucose uptake for PET imaging, fluorescent versions of 2-deoxy glucose, such as IRDye 800CW 2-deoxy glucose conjugate (2-DG) have also been developed 28. Despite the controversial study claiming that NIR 2-DG probes are accumulated non-specifically in tumors and as such are not a substitute for FDG 29, NIR optical metabolic probes still hold a great potential when visualized using adequate instruments capable of imaging in the NIR range, both in microscopy and macroscopy. NIR 2-DG probes are critical to implement a multiplexed approach that quantifies both metabolic status and intracellular drug delivery via FLI FRET in preclinical cancer models. This is important not only for monitoring drug response in real time, but also for potential indication of drug delivery problems since glycolytic tumors tend to have poor blood perfusion 30-32.

Importantly, for optimal FRET pair performance, the donor emission and acceptor excitation spectra need to have a large spectral overlap along with enough separation to avoid signal crosstalk. These requirements render multiplexing FRET with other fluorescence intensity signals particularly difficult to achieve. Recent development of dark quenchers 33-35, which do not produce inherent fluorescence emission but still undergo quenching via several mechanisms, including FRET and static quenching, has been harnessed to mitigate high signal crosstalk and allow multiplexing FRET pairs with other fluorophores. These quenchers have been primarily designed for visible fluorophores, but some quenchers such as black hole quencher-3 (BHQ-3) and SiNQs 34 have been developed for longer-wavelength probes. However, the application of these quenchers is limited due to their relatively narrow spectrum of absorbance. Conversely, IRDye QC-1 (QC-1; Li-Cor, Inc.) is a dark quencher with a much broader absorbance spectrum that encompasses the NIR and visible range 36,37. To date, QC-1 has been used as a quencher in activatable probes in imaging of antibody internalization 38,39, enzymatic activity 40, and as a marker for photoacoustic imaging 41-43. Yet, the characterization of the binding of QC-1- conjugated protein ligands to their respective receptors in cell-based assays as well as of the QC-1 behavior as an acceptor in FLI-FRET applications *in vitro* or *in vivo* is severely lacking.

To the best of our knowledge, this is the first report of QC-1 as an acceptor for NIR FLI-FRET, using the transferrin (Tf)-transferrin receptor (TfR) system as a biological model for drug delivery in live intact animals. The multiplexed imaging of glucose metabolism and receptor-ligand target engagement was achieved using Tf-QC-1 conjugates together with 2-DG probes in the MFLI-FRET imaging of breast tumor xenografts.

## Experimental Methods

### Ligand labeling

Human holo Tf (Sigma) was conjugated to Alexa Fluor 700 (AF700) or AF750 (Life Technologies) through monoreactive N-hydroxysuccinimide ester to lysine residues in the presence of 100 mM sodium bicarbonate, pH 8.3, according to manufacturer's instructions 14. The probes were purified using Amicon Ultra-4 centrifugal filter units (MWCO 30 kDa). After extensive washes with phosphate buffered saline (PBS), the probes were reconstituted in PBS and protein concentration was normalized to 1 mg/mL 14,22. The degree of labeling of the probes was assessed by spectrophotometer DU 640 (Beckman Coulter, Fullerton, CA, USA). The average degree of labeling was no more than 2 fluorophores per Tf molecule. Custom Tf-QC-1 conjugation was performed by Li-Cor (Lincoln, NB, USA) with average dye to protein ratio of 3. All ligands were normalized to concentration 1 mg/mL in phosphate-buffered saline pH 7.6 and filter sterilized. In **Figure S1**, several fluorescently labeled or unlabeled Tf probes, kept at 4 °C for 2-6 weeks since conjugation, were subjected to immunoblotting analysis using anti-human Tf. All Tf probes displayed overall good protein stability when stored at 4 °C, for 2-6 weeks.

### Hyperspectral imaging

AF700 conjugated to murine IgG (MG129, Thermo Fisher Scientific, MA) was combined with goat anti-mouse secondary antibody conjugated to AF750 (A-21037, Thermo Fisher Scientific) or QC-1 (custom made by Li-Cor), at different Acceptor : Donor (A:D) concentration ratios. While keeping the donor concentration constant at 50 µg/mL, the multi-well plate samples contained respective ratio A:D of 0:1, 1:1, 2:1, 3:1, 1:0, 2:0 and 3:0. PBS was used as the solvent and two out of the nine total wells were filled only with PBS to act as negative controls. The sample was imaged using a single-pixel hyperspectral fluorescence lifetime imaging system 44. The sample was excited at 695 nm using a Mai Tai HP (High-Performance, Mode-Locked, Ti-Sapphire Laser with a repetition rate of 80 MHz).

The system is composed of structured illumination and detection through a DMD arrangement and is capable of hyperspectrally describing the mentioned sample through the use of PMT-TCSPC based detection. The 16-channel PMT allows for hyperspectral detection ranging from 715 to 780 nm. Each detection channel is approximately 4.5 nm apart of each other. A 715 nm long pass filter (Semrock, FF01-715/LP-25) was used to remove the excitation illumination from the detected signal. The sample was exposed for 1.5 s per pattern and a total of 512 frequency arranged Hadamard patterns were acquired to reconstruct 64×64 resolution intensity and lifetime images. Continuous wave fluorescence intensity and time-domain reconstructions were retrieved using TVAL3 44. Continuous wave fluorescence intensity was obtained by integrating over time the fluorescence decays at each emission channel collected after excitation of the samples with a pulsed laser, whereas the lifetime maps were obtained following the fitting procedure described previously.

### Wide-field macroscopic fluorescence lifetime imaging platform

MFLI was performed on a wide-field time-domain fluorescence lifetime imaging tomographic system, as described previously 13,14,22,24,45,46. Briefly, the system excitation source was a tunable Ti-Sapphire laser (Mai Tai HP, Spectra-Physics, CA). The spectral range was 690 - 1040 nm with 100 fs pulses at 80 MHz. The laser was coupled to a digital micro-mirror (DMD) device (DLi 4110, Texas Instruments, TX), which produced a wide-field illumination over an 8×6 cm area with 256 grayscale levels encoding at each pixel. The wide-field illumination was spatially modulated by controlling the DMD via Microsoft PowerPoint to ensure optimal signal detection over the whole animal body 47,48. The detection system was a time-gated intensified CCD (ICCD) camera (Picostar HR, Lavision GmbH, Germany). The gate width on the ICCD camera was set to 300 ps. The Instrument Response Function (IRF) and fluorescence signals were collected with 40 ps time interval over a 2.0 ns time window and a 7.0 ns time window, respectively. The total number of gates acquired was 175 and the maximum range of detection was 4096 photons per pixel per gate. The multichannel plate gain (MCP) employed for signal amplification was set to 550 V for the whole imaging session. In this study, imaging was performed in reflectance mode 24,46. The laser excitation for AF700 was set at 695 nm and the emission filters were 720±6.5 nm (FF01-720/13-25, Semrock, IL) and 715 nm long pass filter (Semrock, FF01-715/LP-25). The laser for IRDye 800CW was set to 750 or 695 nm and the emission filters were 820±6 nm (Semrock, FF01-820/12-25) and 810±45 nm (Chroma Technology, ET810/90). The IRF was measured by using a full-field pattern projected on diffuse white paper and acquiring the temporal point spread function (TPSF) without using an emission filter. The imaging platform was equipped with an isoflurane anesthesia machine and a warming device, as described in 14,46. Upon different periods of time post-injection (p.i.), animals were imaged using MFLI platform. The imaging parameters were identical for all the mice.

### Cell culture

Breast cancer cells T47D, MDA-MB-231, SKBR3 and epithelial cells MCF10A were obtained from ATCC (Manassas, VA, USA). Cancer cells were cultured in Dulbecco's modified Eagle's medium (Life Technologies) supplemented with 10% fetal calf serum (ATCC), 4 mM L-glutamine (Life Technologies), 10 mM HEPES (Sigma) and Penicillin/Streptomycin (50 Units/mL/50 µg/mL, Life Technologies) at 37 °C and 5% CO_2_. MCF10A cells were cultured in DMEM/F12 with 5% horse serum, 20 ng/ml EGF, 0.5 mg/mL hydrocortisone, 100 ng/ml cholera toxin, 10 µg/mL bovine insulin and penicillin/streptomycin.

### Tf-QC-1 vs Tf-AF700 internalization in cancer cells

For *in vitro* uptake experiment, T47D cells were plated at 400,000 cells per plate, on MatTek 35 mm glass bottom plate (Ashland, MA), and cultured overnight followed by 30 min incubation with DHB imaging medium (phenol red-free DMEM, 5 mg/mL bovine serum albumin (Sigma), 4 mM L-glutamine, 20 mM HEPES (Sigma) pH 7.4). After that the cells were incubated for 1 h at 37 °C with 40 µg/mL either Tf-AF700 or Tf-QC-1 diluted in DHB solution. Tf internalization was stopped by washing the cells with cold HBSS buffer (Life Technologies), followed by fixing for 15 min with 4% paraformaldehyde (PFA) and processing the samples for immunocytochemistry using mouse monoclonal Tf antibody (Serotech 9100-1057) and rabbit polyclonal TfR antibody (Abcam ab84036) diluted 1:250. Secondary antibodies F(ab) goat anti mouse AF555 and goat anti rabbit AF488 (Life Technologies) were used at 1:500 dilution. Samples were imaged on Zeiss LSM 880 confocal microscope using the same imaging settings for all samples. Collected z-stacks were analyzed pixel by pixel for colocalization using Pearson's coefficient via Imaris software (Bitplane).

### NIR FLIM FRET microscopy

T47D breast cancer cells were plated on MatTek 35 mm glass bottom plates and cultured overnight. Cells were washed with HBSS buffer, precleared for 30 min with DHB imaging medium followed by incubation with Tf FRET pair probes (Tf-AF700, Tf-AF750 or Tf-QC-1), for 1 h at 37 °C, at Acceptor : Donor ratio (A:D) 0:1, 0.5:1, 1:1, 2:1 and 3:1, with Tf-AF700 donor concentration constant at 20 µg/mL. After Tf internalization, cells were washed with cold HBSS buffer, fixed with 4% PFA, and stored in DHB solution for imaging. NIR FLIM FRET microscopy was performed on a Zeiss LSM 880 Airyscan NLO multiphoton confocal microscope using an HPM-100-40 high speed hybrid FLIM detector (GaAs 300-730 nm; Becker & Hickl) and a Titanium: Sapphire laser (Ti: Sa) (680-1040 nm; Chameleon Ultra II, Coherent, Inc.) (**Figure S2**). The Ti: Sa laser was used in conventional one-photon excitation mode. Because of this, the FLIM detector was attached to the confocal output of the scan head. On the LSM 880 with Airyscan, the confocal output from the scan head is used for the 'Airy-Scan' detector and thus it is not directly accessible. However, to accommodate the HPM-100-40 detector a Zeiss switching mirror was inserted between the scan head and the Airyscan detector. The 90° position of the switching mirror directs the beam to a vertical port to which the FLIM detector was attached via a Becker & Hickl beamsplitter assembly. A Semrock FF01-716/40 band pass filter and a Semrock FF01-715/LP blocking edge long-pass filter were inserted in the beamsplitter assembly to detect the emission from AF700 and to block scattered light, respectively. The 80/20 beamsplitter in the internal beamsplitter wheel in the LSM 880 was used to direct the 690 nm excitation light to the sample and to pass the emission fluorescence to the FLIM detector. An excitation wavelength of 690 nm was selected for cells incubated with Tf-AF700 (donor specimen), Tf-AF700 and Tf-AF750 (donor plus acceptor sample) or Tf-AF700 and Tf-QC-1 (donor plus dark quencher acceptor); at this wavelength, cells incubated with only Tf-AF750 showed negligible intensity levels. The amount of donor photobleaching was monitored by measuring intensity changes over time. Donor photobleaching levels were reduced (<5%) at the 690 nm excitation power level used in NIR FLIM-FRET experiments. The data was analyzed by two-component exponential fitting using SPCImage software (Becker & Hickl GmbH, Germany) to determine *A*_1_ and *A*_2_, the amplitudes of short (fraction of donors involved in FRET events) and long (fraction of donors NOT involved in FRET events) donor lifetime components, respectively, and τ_1_ and τ_2_ the quenched and unquenched donor lifetimes. *A*_1_ is henceforth denoted as FRET donor fraction - FD%, as previously 49,50. A χ^2^ fitness test was used to determine the validity of the fit, providing χ^2^ values <1.5 for all pixels.

### Immunoblotting

Whole cell lysates of breast cancer cell lines T47D, MDA-MB-231, SKBR3 and non-tumorigenic epithelial cell line MCF10A were processed for immunoblotting using standard technique as in 14,51. Blots were probed with rabbit polyclonal TfR antibody diluted 1:1000 (Abcam, cat# 84036) and mouse monoclonal β-actin antibody diluted 1:10,000 (Abcam, cat# 8226).

### Animal experiments

All animal procedures were conducted with the approval of the Institutional Animal Care and Use Committee at both Albany Medical College and Rensselaer Polytechnic Institute. Animal facilities of both institutions have been accredited by the American Association for Accreditation for Laboratory Animals Care International. To produce Matrigel plugs, T47D cells were plated in 60 mm dishes and cultured until confluent. The cells were washed with HBSS buffer and precleared with DHB medium for 30 min. The cells were then loaded with Tf labeled with the appropriate FRET pair AF700/QC or AF700/AF750 at A:D ratios of 0:1, 1:1, and 2:1 for 1 h, keeping a 40 µg/mL constant donor concentration. After internalization, the cells were fixed with 4% PFA, scraped, pelleted, and resuspended in Matrigel (Corning). The 100 µL cell samples in Matrigel (1:1 ratio) were injected into the inguinal and thoracic fat pads on both sides of the anesthetized athymic nude mice for both FRET pairs. Matrigel plugs were allowed to solidify for two hours before imaging.

Tumor xenografts were generated by injecting 5×10^6^ T47D cells expressing either GFP (tumors M1 and M2) or ZsGreen (tumor M3) in phosphate-buffered saline (PBS) mixed 1:1 with Cultrex BME (R&D Systems Inc, Minneapolis, MN, USA) into the right inguinal mammary fat pad of female 5-week old athymic nude mice (NU(NCr)-Foxn1^nu^, Charles River Laboratories, Wilmington, MA, USA). The subcutaneous tumors were allowed to grow for 4-5 weeks and were monitored daily. AF700 and Tf-AF750 (20 and 40 µg respectively in the volume 100-120 µL) were injected through the lateral tail vein in animals restrained with DecapiCones. For IRDye 800CW 2-DG (2-DG; Li-Cor) imaging 100 µL of probe (10 nmol/animal) was tail-vein injected in the mice subjected to overnight fasting.

### Immunohistochemistry

After imaging, tumors were excised, fixed in formalin, and paraffin embedded. Epitope retrieval was performed by boiling deparaffinized and rehydrated 5 µm sections in 10 mM Sodium citrate pH 6.0 for 20 min. IHC staining was carried out using a standard protocol from Vectastain ABC Elite kit (Vector Labs, Burlingame, CA; PK-6101). Vector NovaRED (Vector Labs) was used as a peroxidase substrate. Tissue sections were counterstained with Methyl Green (Sigma, M8884). Hematoxylin Eosin stain was used for basic histology. Primary antibodies were as followed: rabbit polyclonal Tf (1:2,000; Abcam; #1223), rabbit polyclonal TfR (1:250; Abcam; #84036), rabbit polyclonal glucose transporter 1 (GLUT1; 1:200) (ThermoFisher Scientific; PA1-46152). Brightfield images were acquired using Olympus BX40 microscope equipped with Infinity 3 camera (Lumenera Inc., Ottawa, ON, Canada). For immunofluorescent staining, 10 µm frozen tumors sections were fixed with 4% paraformaldehyde, processed for microscopy using same rabbit polyclonal TfR antibody (1:250) and goat anti rabbit F(ab) AF555 (1:500) and imaged on Zeiss LSM 880.

### Bi-exponential fitting to extract FRETing donor fraction in MFLI imaging

The FRETing donor fraction within a region of interest (ROI) was quantified by fitting the fluorescence decays in each pixel of ROI to the bi-exponential model:


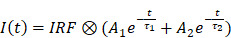
(1)

Here I(t) represents the fluorescence decay and IRF is the instrument response function inherent to the system and collected on a piece of diffuse white paper. Moreover, *A*_1_ and *A*_2_ represent the amplitudes of short (donors involved in FRET events) and long (donors NOT involved in FRET events) donor lifetime components respectively, τ_1_ and τ_2_ are the quenched and unquenched donor lifetimes, t is time, and 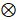 represents convolution. The tail portions of the decays (99-2% of the peak value) in each pixel of an ROI are fit to the bi-exponential model to extract amplitude of short donor lifetime component (*A*_1_, henceforth denoted as FRET donor fraction - FD%) using the Matlab function fmincon for least squares minimization of the cost function 13,24,46. The fluorescence decays from the MFLI system and the hyperspectral system were analyzed with the same fitting parameters and smoothing with Anscombe filtering. The average values and standard deviations are reported.

### Statistical analysis

The statistical significance of the data was tested with Anova repeated measures or unpaired Student's *t* tests as described for each specific experiment (see Supplemental Tables). Differences were considered significant if the *p*-value was less than 0.05. Error bars indicate standard deviation or 95% confidence interval.

## Results and Discussion

### Monitoring target engagement and metabolic levels in tumor xenografts in live mice

The ability to measure the binding and internalization of targeted therapeutics into tumor cells in combination with the detection of metabolic levels in an undisturbed tumor environment could provide fundamental novel information regarding molecular profile, delivery efficacy and metabolic drug response in each individual tumor xenograft. In this study, we capitalized on TfR-Tf binding to quantify target engagement and internalization of ligand-receptor complexes, as shown previously 14,22-25 as well as in 2-DG imaging. 2-DG specifically accumulates in metabolically active tissues with high glycolysis and glucose transporter 1 (GLUT1) levels 52-55. Because of increased iron need due to high levels of proliferation and metabolism, transferrin receptor (TfR) is overexpressed both in cancer cells and tissues 14,18,56. Thus, TfR has been widely used as a target for molecular imaging 57,58. Moreover, Tf, a human serum protein and TfR's native ligand 56, has been utilized as a carrier for targeted drug delivery 59,60. In**Figure S3A**, immunoblotting analysis of MCF10A, a non-cancerous human mammary epithelial cell line, and T47D, MDA-MB-231 and SKBR3 human breast cancer cell lysates using anti-TfR and anti-β-actin, as a loading control, clearly shows that T47D cells display higher levels of TfR expression than other cell types. In **Figure S3B**, we showed immunofluorescence analysis of TfR expression in T47D tumor tissue sections. Overall, TfR expression levels are increased in both T47D cells and tumors (**Figure S3**). T47D breast cancer cell line also overexpresses estrogen receptor and represents the most common type of breast cancer - luminal A 61.

**Figure 1:**
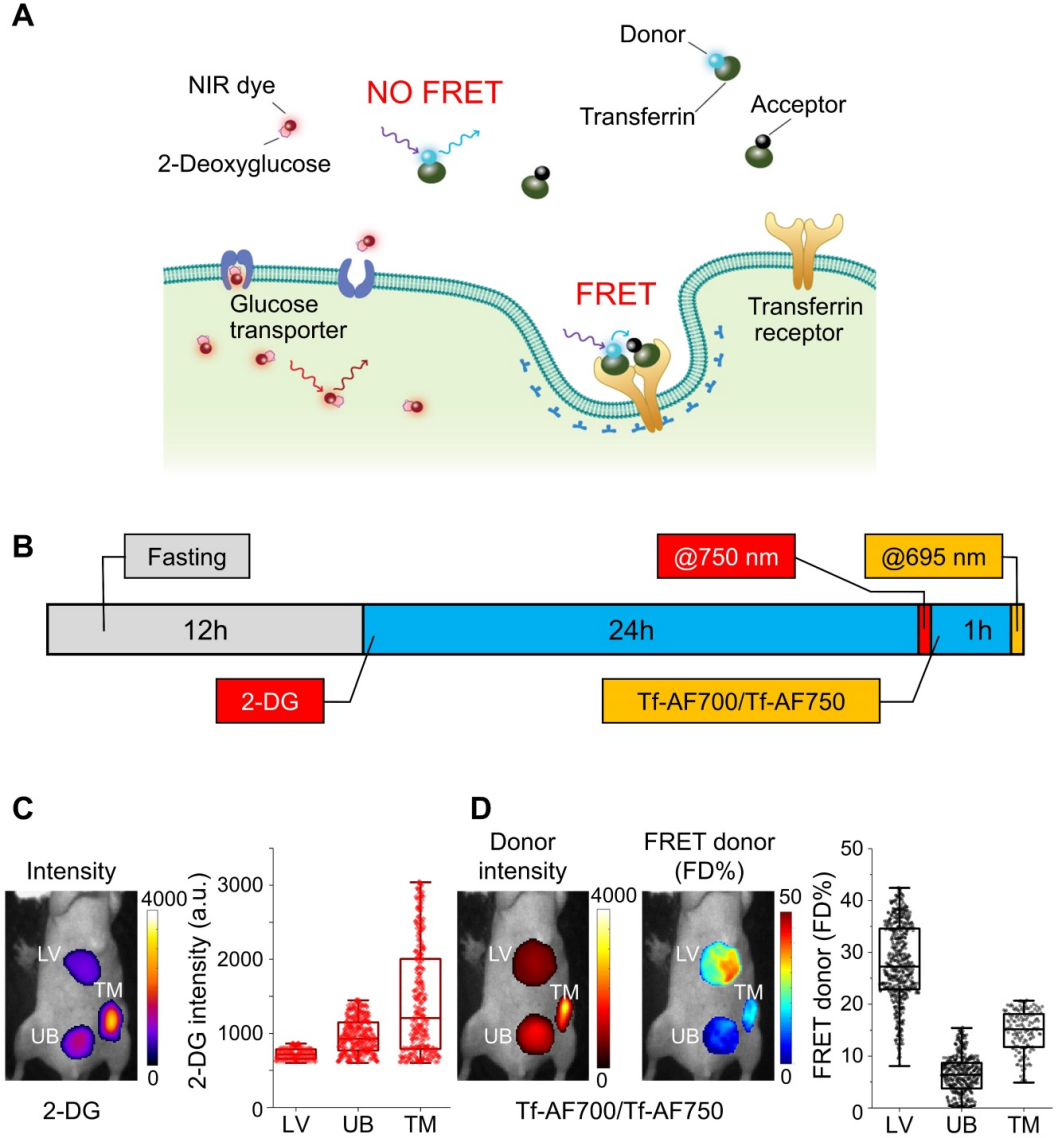
** Multiplexed Forster resonance energy transfer macroscopy fluorescence lifetime imaging (FRET MFLI) and metabolic *in vivo* imaging. (A)** Schematic representation of IRDye 800CW 2-DG (2-DG) based metabolic imaging and measurement of target engagement via FRET-MFLI *in vivo* imaging. FRET signal is detected by the reduction of donor fluorophore lifetime upon binding of donor- and acceptor-labeled Tf ligands to the transferrin-receptor (TfR). 2-DG fluorescence intensity indicates the location of 2-DG molecules, either diffused in extracellular space or internalized in tumor cells via glucose transporter 1 (GLUT1). **(B-D)** Sequential 2-DG and FRET MFLI *in vivo* imaging. **(B)** Imaging protocol includes mouse overnight fasting up to 12 h prior to 2-DG injection and imaging at 24 h post-injection (p.i.), followed by transferrin-Alexa Fluor700 (Tf-AF700)/Tf-AF750 FRET pair (acceptor : donor, A:D = 2:1) injection and imaging 1 h p.i. **(C)** 2-DG fluorescence intensity image (left panel) and respective graph displaying distribution of 2-DG signal per region of interest (ROI) pixel (right panel) of mouse liver (LV), tumor xenograft (TM) and urinary bladder (UB). **(D)** Left panel displays Tf-AF700 intensity (total Tf, including bound and unbound) and FD% (bound Tf) map of liver (LV), tumor (TM) and urinary bladder (UB) in mouse sequentially injected with 2-DG and Tf-AF700/Tf-AF750 and imaged with MFLI at 24 h and 1 h p.i., respectively. Right panel shows distribution of FRET donor fraction (FD%) signal per ROI pixel. Rectangle box indicates 25%-75% pixel values, horizontal line indicates median value and vertical line indicates range within 1.5 quartile. Statistical analysis of FD% and 2-DG values between tumor and bladder using student t-test is shown in **Table S1.**

To monitor metabolic imaging and TfR-Tf internalization in the same T47D breast tumor xenograft, we performed FRET MFLI sequential imaging to measure the fluorescence signal of 2-DG, a NIR-labeled glucose metabolic marker and the Tf-TfR engagement using the Tf-AF700/Tf-AF750 FRET pair, respectively (**Figure 1A**). FRET signal is an indication of the fraction of TfR-bound and internalized Tf ligand (FRET donor fraction; FD%) and is detected by the reduction of donor fluorophore lifetime upon binding of donor- and acceptor-labeled ligand molecules to their respective receptor **(Figure 1A)** 14,22-25. 2-DG fluorescence intensity indicates the accumulation of 2-DG molecules upon transport into tumor cells via GLUT1 (**Figure 1A**) 52-55. However, results from these two imaging approaches are normally compiled from tumor xenografts in different animals, making the preclinical data difficult to interpret, considering the substantial inter-tumor heterogeneity. Here, the goal is to develop an experimental protocol that can collect both 2-DG and FRET MFLI imaging data from the same tumor xenograft. This novel multiplexed imaging methodology would permit precision medicine approach to measure both metabolism and target engagement in the tumor in the same live and intact animal.

An important limitation of multiplexing NIR FRET pairs with NIR dye-protein conjugates is the significant absorption and emission spectra overlap found when combining AF750 acceptor and IRDye 800CW (**Figure S4A & B**). To avoid this crosstalk, MFLI imaging was performed sequentially, as shown in **Figure 1B**. Firstly, mouse fasting was used to increase the signal to noise ratio of 2-DG measurements, which allows for improved tumor delineation and visualization. Secondly, 2-DG tail-vein injection was performed, followed by imaging 24 h p.i at 750 nm excitation. Thirdly, after 2-DG imaging, the NIR-labeled Tf FRET pair at A:D = 2:1 was tail-vein injected and imaged 1 h p.i. at 695 nm excitation. In **Figure 1C & D**, regions of interest (ROIs) highlight different organs in the intact animal subjected to 2-DG, followed by FRET MFLI imaging, as described in **Figure 1B**. Tumor, liver and bladder ROIs, comprising 150-500 pixels, were selected by establishing Tf-AF700 donor fluorescence intensity thresholds. 2-DG accumulated predominantly in the tumor (**Figure 1C**) because of higher level of glycolysis compared to normal tissues. Tumor and liver displayed elevated levels of FD%, indicating binding and internalization of NIR-Tf into TfR-enriched tissues (**Figure 1D**). In contrast, as expected, the urinary bladder showed very low levels of FD%, suggesting that fluorescence signal is due to excreted Tf-AF700 degradation products and free dye 22-24. As shown in **Table S1**, both 2-DG and FRET MFLI signals are significantly increased (p < 0.05) in tumor vs. bladder. Although successful, this imaging experiment was performed using a sequential imaging protocol that increases workload, can lead to registration errors between the spatial mapping of the biomarkers, and may introduce biological bias due to the dynamic nature of tumor biology. Therefore, we investigated the potential of using dark quencher QC-1 as an alternative FRET acceptor to enable multiplexed and simultaneous imaging of each individual live intact animal.

### Characterization of dark quencher IRDye QC-1 as a NIR FRET acceptor

Although QC-1 has been suggested to have broad applicability as an acceptor in FRET assays using a wide variety of fluorescent donor dyes 36, such as AF700 (**Figure S4C**), its use in cell-based FRET imaging assays has been lacking. Moreover, no FLIM imaging of AF700/QC-1 FRET pair has been reported to date. Therefore, we investigated the spectral characteristics of the FRET pair AF700/QC-1 in comparison with our standard AF700/AF750 FRET pair using a hyperspectral single-pixel imaging system with lifetime capabilities 44 to visualize donor and acceptor emission spectra as well as quantify FRET lifetime parameters (**Figure S5**). To this end, we performed an antibody binding FRET multi-well assay, in which donor samples are composed of AF700 conjugated to murine IgG primary antibody (IgG-AF700) and acceptor samples include goat anti-mouse secondary antibody conjugated to QC-1 (anti-IgG-QC-1) or AF750 (anti-IgG-AF750), at various A:D ratios.

As expected, upon excitation at 695 nm, the wells containing AF700 and QC-1 or AF750 at A:D ratios from 0:1 to 3:1, displayed a decreasing AF700 fluorescence intensity as the QC-1 (**Figure S5A & B**) or AF750 (**Figure S5D & E**) concentrations increased. Importantly, acceptor bleed-through was observed for AF750 (**Figure S5E**) but not for QC-1 (**Figure S5B**), indicating that bleed-through from direct excitation of acceptor fluorophores by the illumination that is used to excite the donor and produce FRET, occurs for AF750 but not for QC-1. The reconstructed fluorescence lifetime maps show that donor lifetime decreases with increasing acceptor concentration in A:D ratios 1:1, 2:1 and 3:1 due to elevated FRET events (**Figure S5C & F**). Significantly, unlike QC-1, contribution from the AF750 acceptor fluorescence leads to substantial reduction in lifetime values of FRETing AF700/AF750 samples and could be confirmed by the presence of AF750 fluorescence in late spectral channels (> 738 nm). QC-1's outstanding quencher properties minimized the spectral crosstalk, and thus improved the accuracy of the lifetime result, allowing for direct quantification of AF700 FRETing donor fraction and mean lifetimes even in the absence of specific emission filters or spectral unmixing.

**Figure 2:**
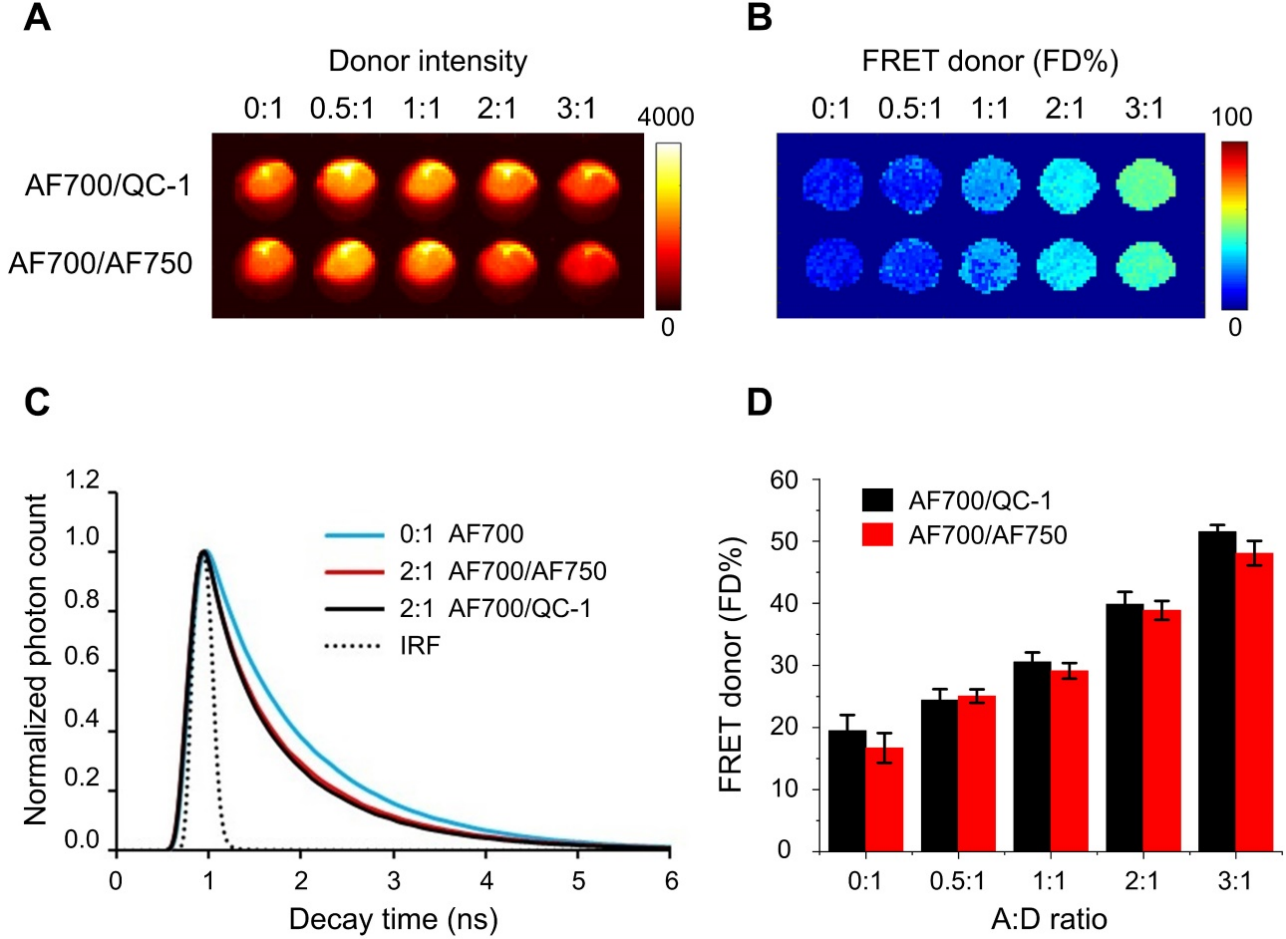
Comparison of AF700/QC1 vs. AF700/AF750 FRET pairs using IgG conjugates in wide-field macroscopy fluorescence lifetime (MFLI) imager. Fluorescence intensity **(A)** and FRET donor fraction (FD%) **(B)** maps of AF700/AF750 and AF700/QC1 FRET samples. **(C)** Donor fluorescence lifetime decay of AF700/AF750 (red line) and AF700/QC1 (black line) samples with acceptor: donor (A:D) ratio 0:1 and 2:1; IRF is instrument response function. **(D)** Quantification of FD% vs A:D ratio for AF700/AF750 (red) and AF700/QC-1 (black) antibody binding FRET assay. Data presented as a mean ± standard deviation. Statistical analysis of AF700/QC1 vs. AF700/AF750 FRET pairs at increasing A:D ratios (not significant; *p* > 0.05) using repeated measures Anova is described in **Table S2.**

Next step was testing QC-1 as the acceptor in a similar antibody binding FRET assay using wide-field time-resolved MFLI imaging platform, as used for small animal imaging 22,25 (**Figure 2**). Fluorescence intensity map (**Figure 2A**) and FD% map (**Figure 2B**) of both AF700/QC-1 and AF700/AF750 IgG samples show decrease of donor fluorescence (quenching) and rise of FD%, respectively, as A:D ratio increases. Fluorescence decays at A:D = 2:1 look similar between AF700/QC-1 and AF700/AF750 IgG samples (**Figure 2C**) and FD% quantification plotted against the A:D ratio (**Figure 2D**) shows that dark quencher acceptor QC-1 performs at similar levels to that of the emitting acceptor AF750 (statistical analysis shown in **Table S2;** *p* > 0.05). Thus, we demonstrated that QC-1 acts as an adequate FRET acceptor when compared to AF750 using either hyperspectral imaging or FRET MFLI wide-field imaging in *in vitro* settings.

### QC-1 as an acceptor in FRET cell-based assays at multiscale

**Figure 3:**
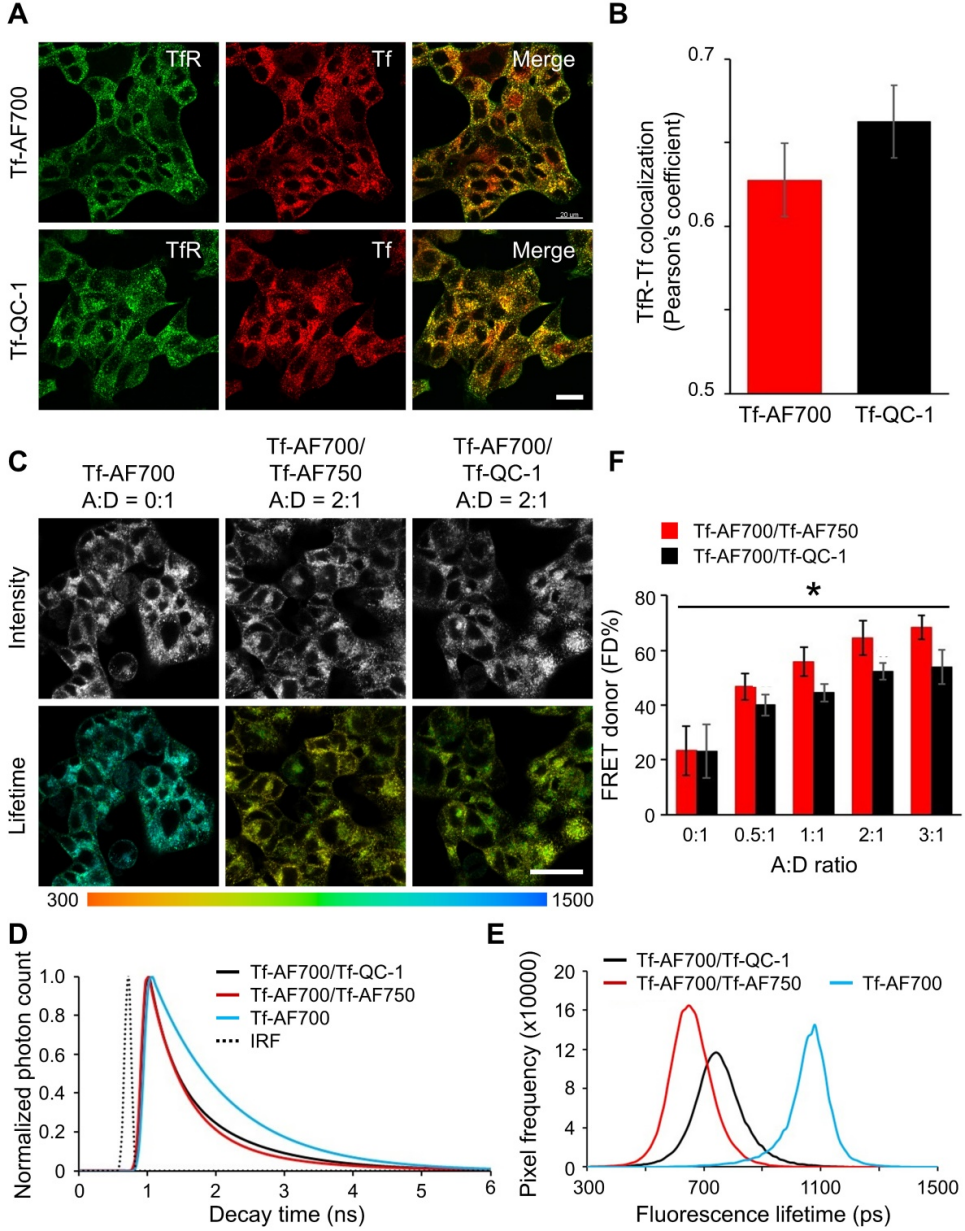
** (A-B)** Transferrin-Alexa Fluor700 (Tf-AF700) and Tf-QC-1 internalization and trafficking in cancer cells. **(A)** Maximum intensity projections of z-stacks consisting of 10-12 optical slices of T47D human cancer cells loaded with Tf-AF700 or Tf-QC-1 and processed for anti-transferrin receptor (TfR; green) and anti-Tf (red) immunostaining. Scale bar = 20 µm. **(B)** Quantification of TfR and Tf colocalization analysis using Pearson's correlation coefficient throughout the entire z-stack using Imaris imaging analysis software. Data is presented as a mean of five independent region of interests (ROIs) from n=5 confocal z-stacks. Error bars represent standard deviation. Statistical analysis of colocalization between TfR and Tf-AF700 or Tf-QC-1, as indicated by Pearson's coefficient (not significant; *p* > 0.05) using two-tailed t-test is shown in **Table S3. (C-F)** Near infrared (NIR) Forster resonance energy transfer fluorescence lifetime microscopy (FRET FLIM) assay in cancer cells using dark quencher acceptor transferrin-QC-1 (Tf-QC-1) conjugates. **(C)** NIR FLIM time-correlated single photon counting (TCSPC) data. The representative images of fluorescence intensity and lifetime map (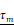; mean lifetime) in T47D cells treated with transferrin-Alexa Fluor700 (Tf-AF700) (acceptor: donor, A:D=0:1), Tf-AF700 and Tf-AF750 (A:D=2:1) or Tf-AF700 and Tf-QC-1 (A:D=2:1); pseudo-color range= 300-1,500 ps. Both fluorescence intensity and lifetime distributions show punctate endocytic structures containing transferrin receptor (TfR)-Tf complexes. Scale bar= 50 µm. (D) Representative fitting curves and instrument response function (IRF), the fluorescent lifetime decay in the single and double-labeled cells was determined by comparing the fitting of the decay data using both single- and double-exponential decay models. **(E)** Comparison of fluorescent lifetime distribution in T47D cells treated with Tf-AF700 (A:D=0:1), Tf-AF700 and Tf-AF750 (A:D=2:1) or Tf-AF700 and Tf-QC1 (A:D=2:1). **(F)** Comparison of FRET donor fraction (FD%) levels in T47D cells treated with Tf-AF700/Tf-AF750 or Tf-AF700/Tf-QC1 FRET pairs at various A:D ratios. Analysis was performed using 10 distinct pixel coordinates from 5 independent region of interests (ROIs). Error bars represent standard deviation. Statistical analysis of Tf-AF700/Tf-QC1 vs. Tf-AF700/Tf-AF750 FRET pairs at increasing A:D ratios (significant; *p* < 0.05) using repeated measures Anova is described in **Table S4.**

To verify that Tf-QC-1 undergoes receptor-mediated endocytosis similarly to previously characterized Tf-fluorophore conjugates in relevant biological settings, we subjected T47D human breast cancer cells to Tf uptake imaging *in vitro* assay by incubation with 40 µg/mL Tf- AF700 or Tf-QC-1 followed by immunostaining using anti-Tf and anti-TfR. Colocalization fluorescence microscopy approach is a standard method to assay the internalization and trafficking of receptor-ligand complexes in adherent cancer cells 51,62. Thus, a fluorescence colocalization confocal microscopy assay was implemented to validate the use of Tf-QC1 vs. standard Tf-AF700 probes for TfR-Tf target engagement. Z-stacks were collected on confocal microscope and analyzed using Imaris software to calculate Pearson's correlation coefficient (**Figure 3A**). Since QC-1 contains an aromatized ring system at its center that increases the rigidity of the structure, it is important to demonstrate that its conjugation to Tf does not affect its interaction with TfR. In case Tf-QC-1 binds TfR less efficiently, the degree of TfR/Tf-QC-1 colocalization would be less in comparison to that of TfR/Tf-AF700. No significant difference (**Table S3**, *p* > 0.05) was detected between TfR and Tf-AF700 or Tf-QC-1 colocalization, as indicated by Pearson's coefficient (**Figure 3B**). These results confirmed that both ligands are internalized and trafficked in a similar manner via receptor-mediated endocytic pathway, suggesting Tf-QC-1 as an adequate probe to follow target engagement of TfR *in vitro* and *in vivo* using NIR FRET FLIM or MFLI imaging, respectively. Additionally, efficient Tf antibody binding to internalized fluorescently labeled human Tf ligands confirmed the biological identity of NIR-labeled Tf probes.

In order to validate QC-1 as an acceptor for FRET at multiscale in the NIR range, we performed NIR FLIM FRET microscopy on Tf uptake assays in T47D cells using Tf-AF700/Tf-AF750 and Tf-AF700/Tf-QC-1 ligands at various A:D ratios. Firstly, cells were imaged using NIR FLIM microscopy system (**Figure S2**) and analyzed with SPCImage (Becker & Hickl). **Figure 3C** shows that both FRET pair ligands demonstrate the same pattern of distribution within the cells. Likewise, both samples had significantly shorter lifetime compared to single donor sample in fluorescence decay graphs (**Figure 3D**) and frequency distribution (**Figure 3E**), as well as linear trend of elevated FD% with increasing A:D ratio (**Figure 3F**). However, in contrast to antibody binding experiment (**Figure 2D**), a small, but statistically significant decrease in FD% levels, was observed in Tf-AF700/Tf-QC-1 cell samples when compared to that in Tf-AF700/Tf-AF750 cells using repeated measures Anova statistics test (**Table S4**; *p* < 0.05).

**Figure 4:**
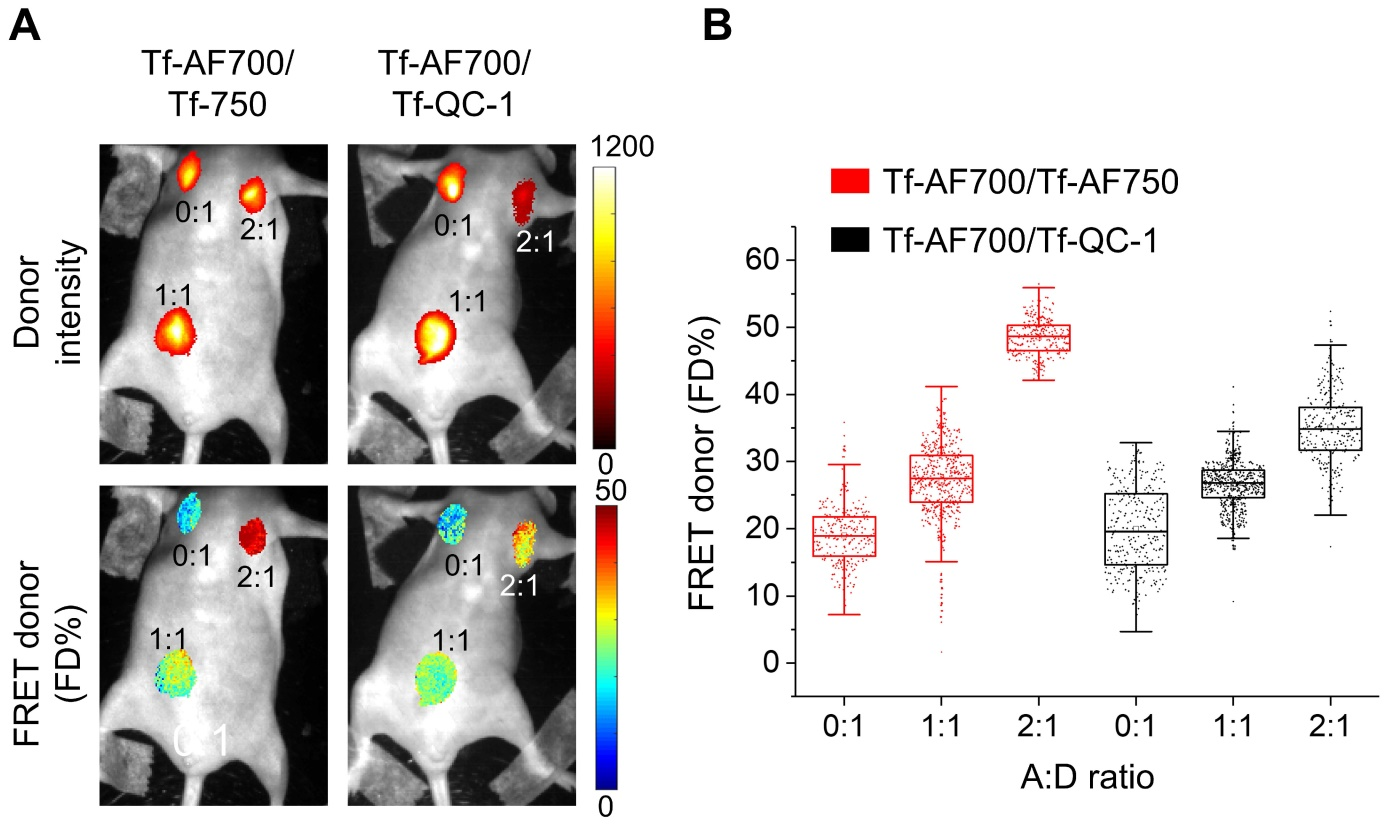
Near infrared (NIR) Forster resonance energy transfer macroscopy fluorescence lifetime imaging (FRET MFLI) of transferrin-QC-1 (Tf-QC-1) as an acceptor in live intact animals. **(A)** Intensity and FRET donor fraction (FD%) maps in Matrigel plugs containing T47D cancer cells preloaded with Tf-AF700/Tf-AF750 or Tf-AF700/Tf-QC-1 ligands with indicated acceptor: donor (A:D) ratio. Anesthetized animals were imaged using wide-field MFLI imager. Because of the different size of injections and the localization of the plug on the animal body, significant variation in fluorescence intensity was detected across the different A:D ratios and FRET pairs in Matrigel plugs. **(B)** FRET quantification in cancer cells containing Matrigel plugs *in vivo*: box plots showing pixel distribution of FRET donor fraction at various A:D ratios.

Secondly, QC-1 was tested as a FRET acceptor in live anesthetized mice implanted with Matrigel plugs containing cancer cells preloaded with Tf-AF700/Tf-AF750 or Tf-AF700/QC-1 at A:D ratio 0:1, 1:1 and 2:1 using the MFLI imager (**Figure 4**). The goal of this experiment was to compare the relationship between FD% vs A:D ratio using these two NIR FRET pairs as they are imaged through living tissues. With such experimental design, the donor signal was localized strictly to the Matrigel plugs. FRET levels in both mice were comparable, showing rising FD% levels with increasing A:D ratios. Again, less FD% in Tf-AF700/Tf-QC-1 plugs were observed (**Figure 4B**). These results suggest that Tf-QC-1 acts as a suitable FRET acceptor in both cell-based assays using NIR FLIM microscopy (**Figure 3**) and MFLI macroscopy (**Figure 4**).

In addition to cell-based and Matrigel plug-based assays, we evaluated QC-1's performance as a FRET acceptor *in vivo* by intravenously injecting several mice with Tf-AF700/Tf-AF750 or Tf-AF700/Tf-QC-1 probes to monitor NIR-labeled Tf biodistribution and target engagement over a time course of 24 h p.i. Several lines of reasoning suggest that Tf-fluorescently labeled conjugates should not have a negative biosafety impact in injected mice. Firstly, Tf is a natural serum protein used as a carrier in many targeted delivery anti-cancer therapeutics 63. Secondly, fluorescently labeled Tf is a well-known endocytic marker widely used in microscopy cell-based protocols that is available commercially for the *in vivo* imaging of tumor xenografts (https://www.perkinelmer.com/product/transferrin-vivo-750-nev10091). Thirdly, QC-1 has been used for multi-spectral optoacoustic diagnoses of breast cancer in pre-clinical models 43. Fourthly, fluorescently labeled or radiolabeled Tf is not associated with adverse reactions in injected animals even during longitudinal imaging studies, with multiple rounds of injections 22,57,64.

**Figure 5:**
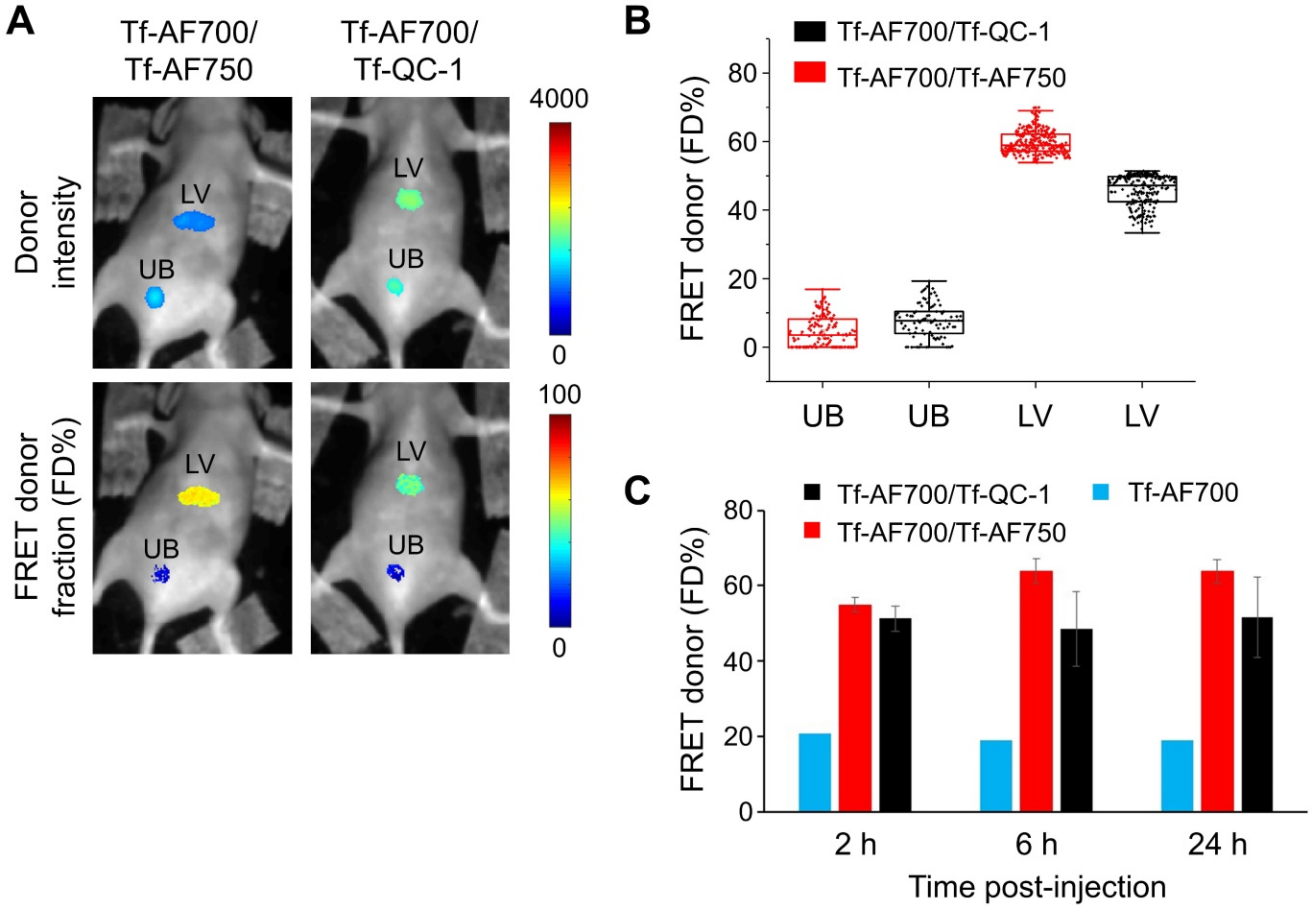
Whole-body Forster resonance energy transfer macroscopy fluorescence lifetime imaging (FRET MFLI) using dark quencher acceptor QC-1 in live intact animals. Nude mice were tail-vein injected with 40 µg/mL transferrin-Alexa Fluor 700 (Tf-AF700) and 80 µg/mL Tf-AF750 or Tf-QC-1 (acceptor: donor, A:D = 2:1) and imaged using MFLI imager at 2, 6 and 24 h post-injection (p.i.). **(A)** Representative liver (LV) and urinary bladder (UB) region of interest (ROI) images of donor intensity (total Tf, including bound and unbound) and FRET donor fraction (FD%) (bound Tf) levels at 24 h p.i. **(B)** Graph displaying distribution of FD% signal per ROI pixel in livers and bladders at 24 h p.i. **(C)** Time course of Tf uptake in livers of mice injected with Tf-AF700 (n=1), Tf-AF700/Tf-AF750 (n=4) and Tf-AF700/Tf-QC-1 (n=3) from two independent experiments. Error bars represent standard deviation. All mice images are shown in **Figure S6.**

FRET MFLI *in vivo* imaging was primarily focused on liver, a main organ for pharmacokinetic studies, which is also a main site for iron metabolism with highly elevated TfR levels 22,24,65. Urinary bladder, an important excretion organ, was used as a negative control since it is expected to accumulate degradation products and free dye 22,24,65. Substantial amount of FRET signal, representing the fraction of internalized NIR-Tf from injected probe, was detected in the liver, but not in the bladder (**Figure 5**). FD% levels in the liver remained at the same low level in the negative control animal injected with donor only during time course, whereas FD% in the livers of mice injected with either FRET pair increased up to three-fold above the control level (**Figure 5C**). The images of mice used in this graph are displayed in **Figure S6.** Important to mention that across all imaging approaches, *in vitro* and *in vivo*, we detected a low, but noticeable, FD% level of ~20% for samples containing donor only (A:D = 0:1). This is most likely due to the application of a two-component biexponential model to a sample that represents a single-component mono-exponential model (“misfitting”). Nevertheless, addition of acceptor always results in significant increases in FD% as expected due to the increased occurrence of FRET events.

From the previous experiments, we determined that Tf-QC-1 can bind TfR at similar levels to Tf-AF conjugates and TfR-Tf-QC-1 receptor-ligand complexes can be transported adequately via the endosomal pathway in T47D cells. Based on soluble antibody binding assays, dark quencher QC-1 FRET FLIM performance was similar to that of standard acceptor AF750 with the advantage of reduced bleed-through levels. However, Tf-AF700/Tf-QC-1 pair consistently showed relatively less FRET signal when compared to that of Tf-AF700/Tf-AF750 in adherent cancer cell culture using NIR FLIM microscopy (**Figure 3**) as well as in live, intact animals using wide-field MFLI imaging (**Figures 4 & 5**). The most likely reason for this difference is the increased Förster distance (R_0_) value of AF700-AF750 (R_0_= 78.1Å) vs. AF700-QC-1 (R_0_= 69Å) FRET pairs (**Figure S7**). A reduced R_0_ value may decrease the positive impact of receptor clustering on overall FRET signal, as described previously 17,66,67. Moreover, *in vitro* antibody binding assays are performed in homogeneous solutions, which are far from real life scenario of cells displaying different ligand local concentration gradients and varying membrane-bound receptor density and clustering. QC-1's structural rigidity may also reduce the ability of Tf-QC-1 conjugates to undergo clustering during endocytic internalization and trafficking, which would also have a negative effect on FRET signal, as shown previously for Tf-quantum dot conjugates 66-68. Therefore, these differences, in both the structure of Tf-QC-1 and in the R_0_ of the AF700/QC-1 FRET pair, may decrease the probability of FRET between two neighboring Tf-TfR complexes due to receptor clustering, contributing to reduced FD% levels in cancer cells loaded with Tf-AF700/Tf-QC-1 FRET pair *in vitro* as well as *in vivo*. Nevertheless, overall QC-1 proved to be an efficient acceptor for FRET pair both for *in vitro* and *in vivo* applications using lifetime-based imaging approaches.

### Multiplexed 2-DG and MFLI-FRET *in vivo* whole-body imaging

**Figure 6:**
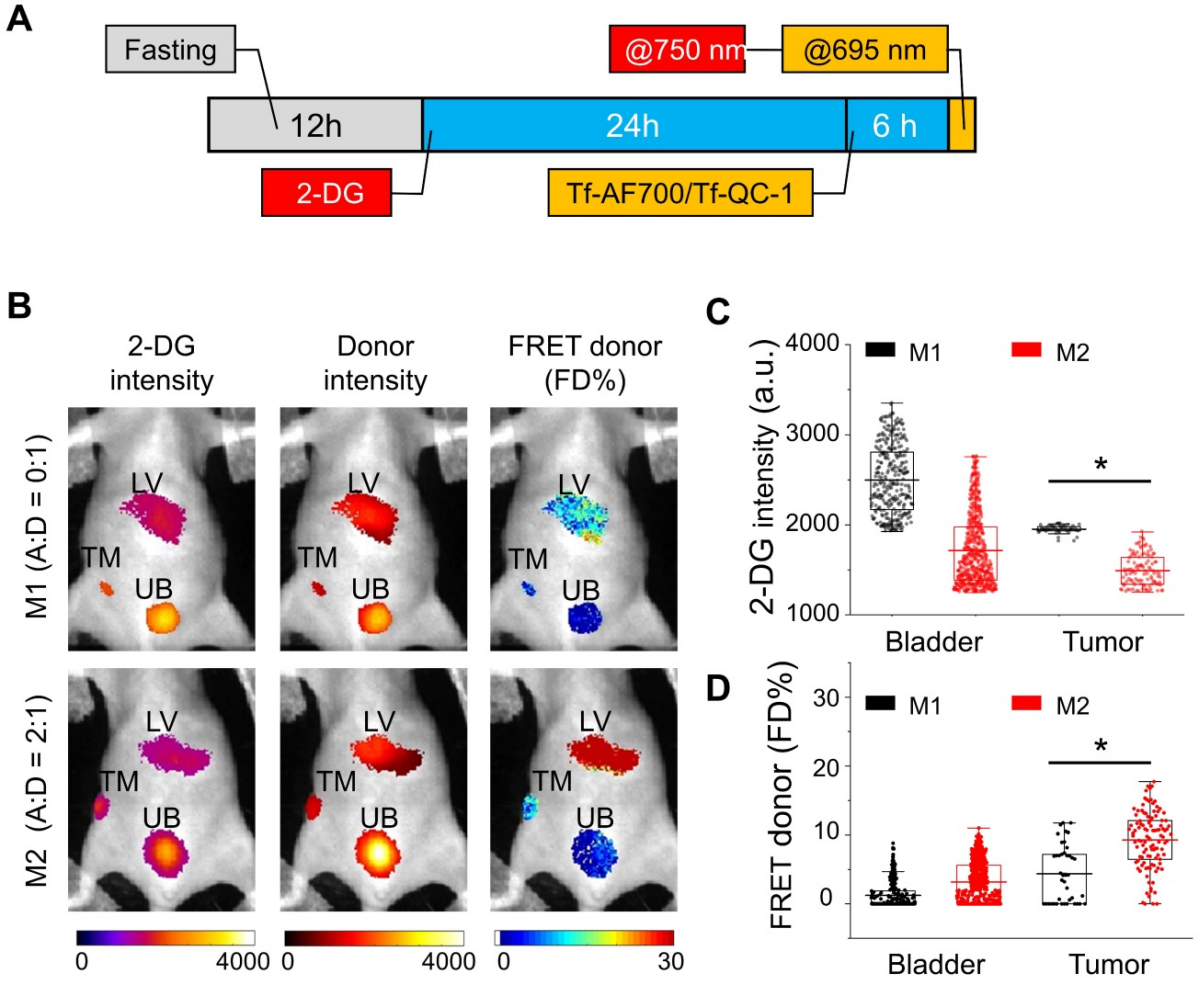
IRDye 800CW 2-DG (2-DG) and Forster resonance energy transfer macroscopy fluorescence lifetime imaging (FRET MFLI) using dark quencher acceptor QC-1 in live intact animals. **(A)** Imaging protocol including fasting, followed by 2-DG and near-infrared transferrin (NIR-Tf) pair injections and MFLI imaging using consecutive imaging at 750 nm and 695 nm excitation steps. **(B)** Region of interest (ROI) images of 2-DG, Tf-AF700 intensity and FRET donor fraction (FD%) levels of liver (LV), tumor (TM) and urinary bladder (UB) in mice injected with 2-DG and Tf-AF700/Tf-QC-1 and imaged with FRET MFLI at 6 h post injection (p.i.). **(C)** Distribution of 2-DG fluorescence intensity per ROI pixel in tumors and bladders. **(D)** Distribution of FD% signal per ROI pixel in tumors and bladders. Asterisks indicate *p*<0.05 (significant). Statistical analysis is presented in Table S5.

The ability to simultaneously image a FRET pair together with 2-DG probe in the NIR range can be achieved when using QC-1 as acceptor, since AF750 excitation and emission spectra significantly overlap with those of IRDye 800CW (**Figure S4**). In this pilot study, we developed an imaging protocol to assess tumor metabolism and drug-target engagement across the same undisturbed whole tumor in a live intact animal. **Figure 6A** displays the imaging protocol, in which animals were subjected to a 24 h fasting period for improved visualization of 2-DG signal, followed by two staggered injections of 2-DG and NIR Tf FRET pair and a quasi-simultaneous imaging session. 2-DG and Tf-AF700/Tf-QC-1 imaging steps were performed in a consecutive manner: 2-DG was excited at 750 nm followed by AF700 excitation at 695 nm. For FRET MFLI imaging, nude mice carrying T47D tumor xenografts were injected with donor only Tf-AF700, A:D = 0:1 (M1 mouse) as a negative FRET control or with Tf-AF750 and Tf-QC-1 at A:D = 2:1 (M2 mouse). As shown in **Figure 6C & D**, tumor, liver and bladder ROIs were selected by establishing Tf-AF700 donor fluorescence intensity thresholds. Tumor's, urinary bladder's as well as liver's ROIs were successfully visualized at 6 h p.i. by sensing the accumulated 2-DG probe and detectable FRET signal from internalized Tf-AF700/Tf-QC-1 (**Figure 6B**). As expected, due to the lack of FRET acceptor, M1 mouse displayed minimal FRET signal in the tumor region in comparison to the M2 mouse (**Figure 6C**). Surprisingly, 2-DG signal, which specifically accumulates in metabolically active tissues with elevated level of glycolysis and GLUT1 52,53, was significantly higher in the M1 tumor than in M2 tumor (**Figure 6D**), despite its smaller size (32 mm^3^).

**Figure 7:**
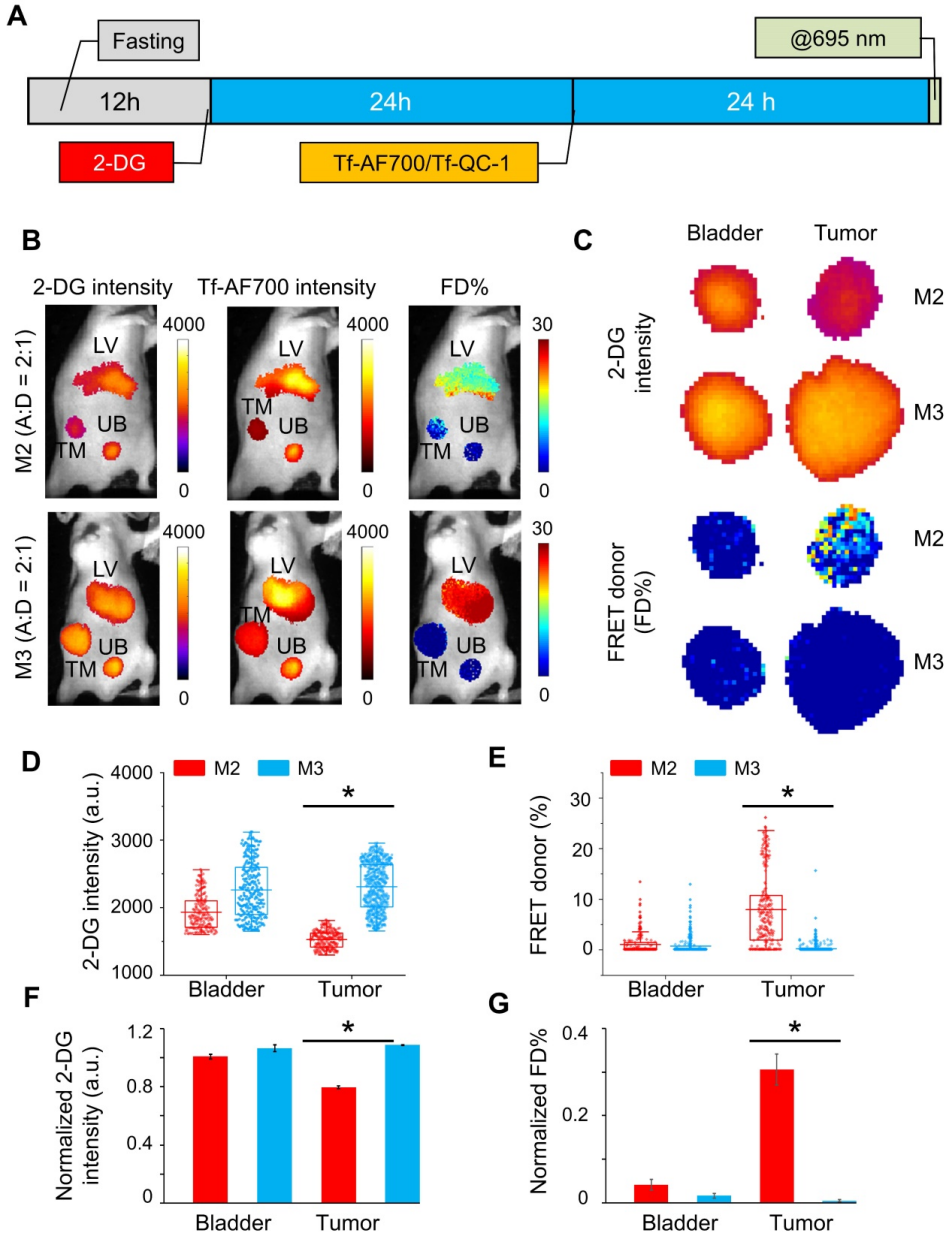
Simultaneous IRDye 800CW 2-DG (2-DG) and Forster resonance energy transfer macroscopy fluorescence lifetime imaging (FRET MFLI) using dark quencher acceptor QC-1 in live intact animals: **(A)** Imaging protocol including fasting, followed by 2-DG and near-infrared transferrin (NIR-Tf) pair injections and MFLI imaging step at 695 nm excitation. **(B)** Images of 2-DG, Tf-AF700 intensity and FRET donor fraction (FD%) levels of liver (LV), tumor (TM) and urinary bladder (UB) in mice injected with 2-DG and Tf-AF700/Tf-QC-1 and simultaneously imaged using FRET MFLI at 24 h post injection (p.i.); single excitation at 695 nm. 2-DG mask was used to determine organ region of interest (ROIs) with the same number of pixels for extraction of FRET and 2-DG data. **(C)** Magnified pixels of 2-DG intensity and FD% in tumors. **(D)** Distribution of 2-DG fluorescence intensity per ROI pixels in tumors and bladders. **(E)** Distribution of FD% signal per ROI pixels in tumors and bladders. Asterisks indicate *p*<0.05 (significant). Statistical analysis is presented in **Table S6. (F)** Graph of normalized to liver values 2-DG intensity and **(G)** FD%. Error bars represent 95% confidence interval, asterisk indicate *p*<0.05 (significant). Statistical analysis is presented in **Table S7.**

In the next experiment, 2-DG signal was used to establish organ ROIs, which can then be applied to correctly extract FRET data for each respective organ. To achieve this, mice were injected with NIR Tf FRET pair after 2-DG injection and imaged simultaneously at 24 h p.i.; both 2-DG and AF700 were excited at 695 nm for 2-DG and FRET data collection, respectively (**Figure 7A**). Then, based on 2-DG signal, fluorescence intensity thresholds were determined to extract tumor, liver and bladder ROIs containing either 2-DG or FRET data. M3 mouse carried a large aggressively growing tumor of 455.5 mm^3^ volume, and M2 mouse was re-used from the previous experiment (within the interval of two weeks), with a tumor size of 98.6 mm^3^. **Figure 7B** shows that both tumors are easily detected by 2-DG signal. However, M3 tumor displayed significantly higher level of 2-DG intensity than M2 tumor (**Figure 7C**). In contrast, an inverse relationship between 2-DG fluorescence intensity and FD% (fraction of receptor bound and internalized ligand) was detected (**Figure 7C-E**). Unlike M3 tumor, the M2 tumor accumulated much less 2-DG but exhibited significantly higher level of Tf-TfR engagement, represented as FRET signal. Stacked bar charts of 2-DG and Tf pixels frequency of both tumors show this inverse relationship in a compelling manner (**Figure S8**). To eliminate the potential variability of injections' volume, we normalized both 2-DG and FRET values to the corresponding values in the liver. Nevertheless, the difference in 2-DG fluorescence intensity between the tumors was still dramatic (**Figure 7F**), whereas FD% levels in the M2 tumor remained much higher than in the M3 tumor (**Figure 7G**).

**Figure 8:**
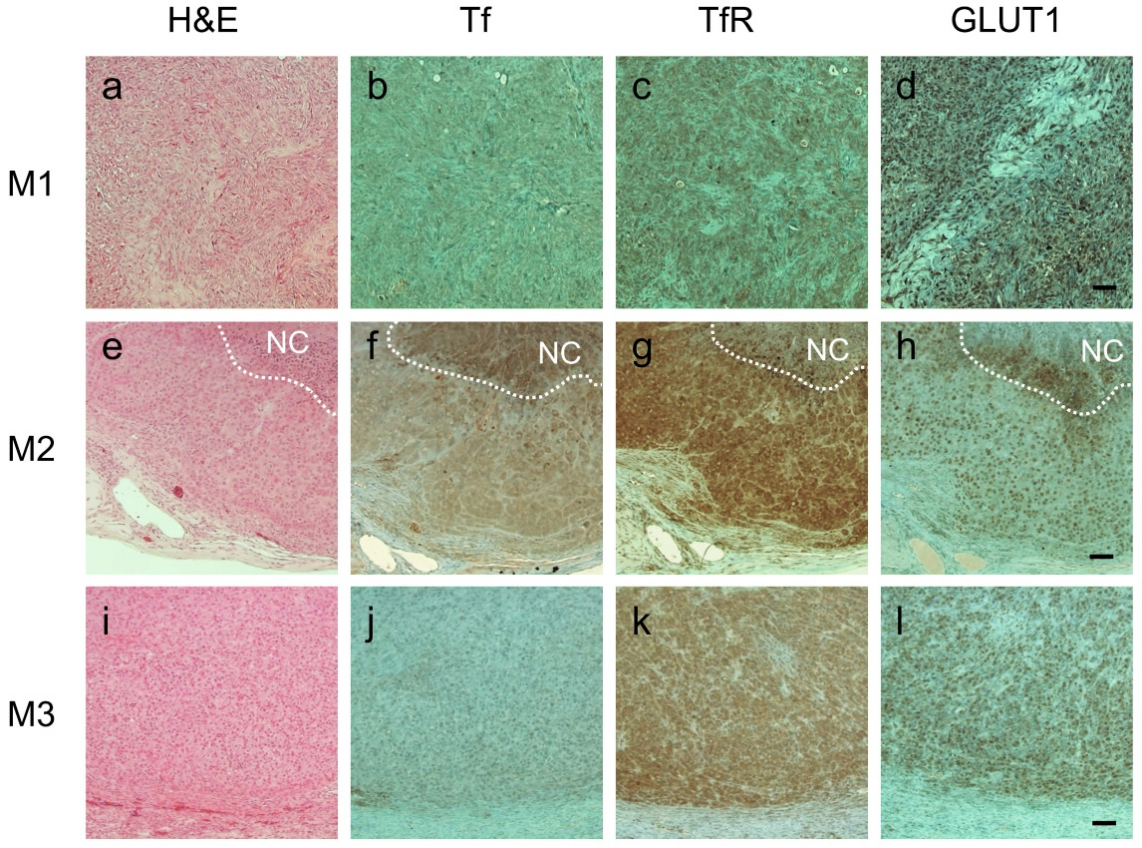
Immunohistochemical staining of consecutive tumor sections from M1 **(A-D)**, M2 **(E-H)** and M3 **(I-L)** tumor xenografts using hematoxylin and eosin **(A, E & I)**, anti-human transferrin (Tf) **(B, F & J)**, anti-human transferrin receptor (TfR) **(C, G & K)** and anti-glucose transporter 1 (GLUT1) **(D, H & I)**. NovaRED was used as peroxidase substrate (brown color), sections were counterstained with methyl green. White dashed lines delineate necrotic core (NC). Scale bar = 100 µm.

Importantly, immunohistochemistry (IHC) staining analysis was in good agreement with FRET imaging data: tumors M1 and M3 had virtually no Tf staining whereas M2 tumor displayed significant number of Tf-positive cells (**Figure 8B, F & J**). All tumors, however, exhibited similarly high levels of TfR (**Figure 8C, G & K**), which is consistent with our previous observation that Tf uptake, correlates with FRET signal, but not with TfR density across the tumors 22. Significantly, IHC staining for GLUT1 matched 2-DG imaging data: M3 and, especially, M1 tumors had visibly higher staining density of GLUT1 compared to M2 tumor (**Figure 8D, H & L**). What makes it particularly relevant is that GLUT1 expression is upregulated by hypoxia-induced factor 1 (HIF-1) and is highly correlated with poor prognosis and survival in most solid tumors 69,70. In agreement with our data, a good correlation between 2-DG signal and GLUT1 IHC staining across the tumors has been shown previously 54,55.

In summary, these results demonstrate for the first time the utility of QC-1 for multiplexed NIR FRET-MFLI imaging in living intact mice carrying tumor xenografts. Two main reasons support the use of dark quencher QC-1 as a FRET acceptor to Tf-AF700 as a FRET donor in the multiplexed 2-DG and FRET *in vivo* imaging of tumor xenografts. Firstly, the use of QC-1 avoids spectral bleed-through between AF750 and IRDye 800 2-DG in the tumor region. Secondly, optical imaging and FRET MFLI in the NIR range are photon-starved techniques. Using QC-1 as a dark quencher acceptor prevents the need of multiple spectral filters to remove potential spectral contamination, thus leading to increased number of photons collected and higher levels of detection sensitivity. Alternatively, unmixing methods could remove the need for QC-1, allowing for effective spectral separation between 2-DG and AF750. Especially, the combination of both spectral and lifetime contrast simultaneously has the promise to enable improved unmixing performances when multiple fluorophores are contributing to the collected emission 71. Though, such approach necessitates advanced imaging systems, such as single-pixel hyperspectral imaging 44 and/or advanced confocal microscopy 66,68,72. Nevertheless, unmixing methods may increase even further the number of biomarkers imaged simultaneously on MFLI.

Importantly, 2-DG imaging, when used in combination with FRET MFLI imaging, is very useful for delineation of tumor margins thus eliminating the reliance on donor intensity-based ROI selection. Unexpectedly, we observed an inverse relationship between 2-DG signal and intracellular Tf accumulation as indicated by Tf FD% signal, despite the differences in experimental design and tumor size. M2 tumor showed increased intracellular accumulation of Tf and reduced expression of GLUT1, both by 2-DG and FRET MFLI imaging as well as IHC staining (**Figure S9**). In contrast, despite their difference in tumor size and growing behavior, both M1 and M3 tumors appeared to possess highest levels of GLUT1 and 2-DG tumor signal (**Figure S9**). Lower Tf delivery can be explained by injection of a reduced amount, as in M1, which was injected with donor probe only, or by poor probe uptake due to aggressive tumor growth behavior, as in M3. In general, aggressively growing tumors have been characterized by poor blood perfusion, stiff extracellular matrix and thus near impenetrable for drug delivery, as well as higher rate of glycolysis and/or GLUT1 density 73,74. These results suggest further studies of a potential relationship between tumor Tf uptake and GLUT1 expression. Moreover, this interesting observation implies that 2-DG metabolic imaging may be indicative of tumor resistance to efficient drug delivery.

Multiplexing *in vivo* imaging of Tf-AF700/Tf-QC-1 FRET signal with NIR 2-DG demonstrates a vast potential for simultaneous monitoring of metabolic response and intracellular drug delivery using MFLI imager. In fact, the reduction of metabolic activity in non-small cell lung cancer by ^18^F-FDG PET imaging has been reported in clinical study as an early prediction of therapeutic response to nivolumab 75. Our technology offers similar but much more straightforward and accessible method with the additional benefit of multiplexing. Here, we demonstrated the use of dark quencher QC-1 as a standard FRET acceptor in NIR lifetime-sensing and, in many cases, the only option for efficient multiplexed FRET imaging in *in vivo* small animal preclinical research. With the advancement of new NIR fluorophores and probes on the market, it is a matter of time to develop a protocol for robust monitoring of polytherapy drug delivery combined with metabolic response in preclinical studies.

## Conclusion

In this study, we developed an advanced protocol for simultaneous quantification of target engagement and monitoring tumor metabolic status via FRET and 2-DG imaging, respectively, in preclinical studies. Such approach was only possible using dark quencher QC-1 as NIR FRET acceptor which we fully characterized on multiscale level *in vitro* and *in vivo*. 2-DG and FRET MFLI multiplexed imaging methodology is essential to visualize and measure drug delivery and metabolic response in undisturbed tumor microenvironment in real time. This novel imaging protocol allows for the establishment of precision medicine approaches, in which multiplexed MFLI imaging measures metabolism and target engagement simultaneously and non-invasively in the same intact tumor xenograft, thus providing a robust means to optimize tumor drug delivery and drastically improve the efficacy of drug development in preclinical studies.

## Supplementary Material

Supplementary figures and tables.
