# Supplementary material for: Multiplexed non-invasive tumor imaging of glucose metabolism and receptor-ligand engagement using dark quencher FRET acceptor
Source: Theranostics. 2020 Aug 15;10(22):10309–25. doi: 10.7150/thno.45825 (PMC7481426; doi:10.7150/thno.45825)
Supplement: Supplementary file 1 — Supplementary figures and tables. [file thnov10p10309s1.pdf]

Supplementary figures

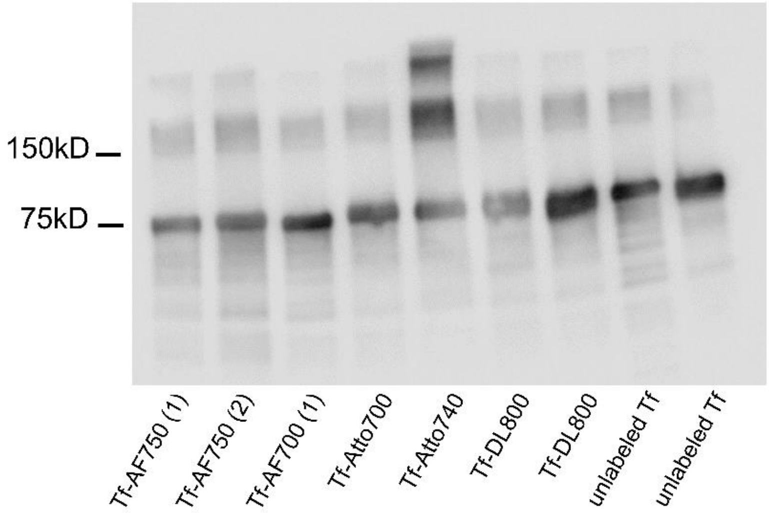

Supplementary Figure 1. Western blot analysis of various fluorescently labeled and unlabeled Tf probes stored for 2-6 weeks since conjugation using antibodies against human Tf. Different near-infrared (NIR) fluorophores were used to generate various transferrin conjugates: Transferrin-Alexa Fluor 750 (Tf-AF750-1 and -2), Tf-Alexa Fluor 700 (Tf-AF700), Tf-Atto700, Tf-Atto740, and Tf-Dylight 800.

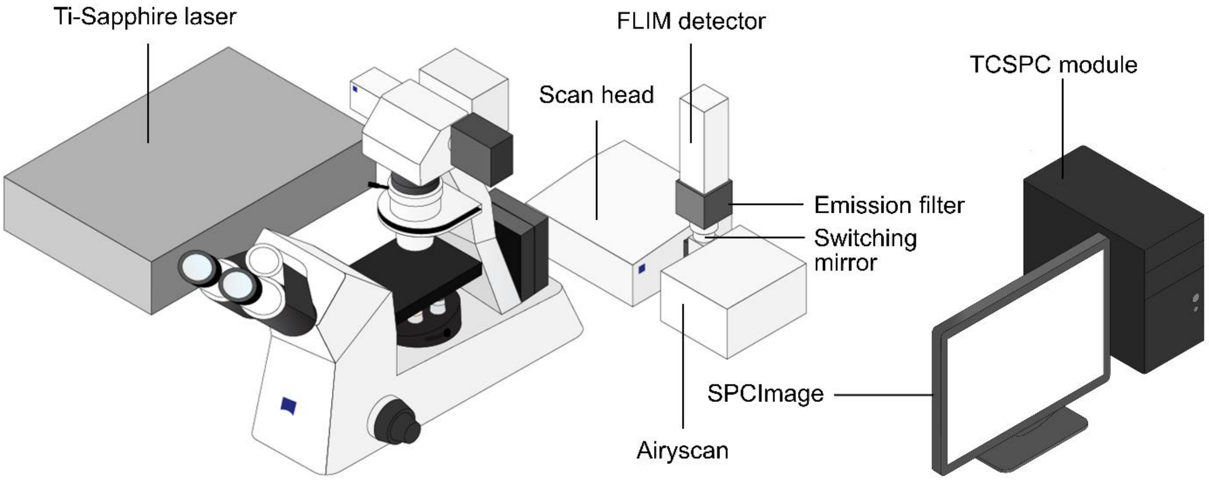

Supplementary Figure 2. Schematic representation of FLIM system on Zeiss LSM880 Airyscan multiphoton microscope. Ti: Sapphire laser (Chameleon) used in conventional one-photon excitation mode; HPM-100-40 high speed hybrid FLIM detector (Becker & Hickl) is directly coupled to the confocal output of the scan head; a Semrock FF01-716/40 band pass filter and a FF01-715/LP blocking edge long-pass filters are inserted in the beamsplitter assembly to detect the emission from AF700 and to block scattered light, respectively. NIR FLIM was performed using the descanned detection pathway via direct connection and a Ti: Sapphire laser as a single-photon excitation source.

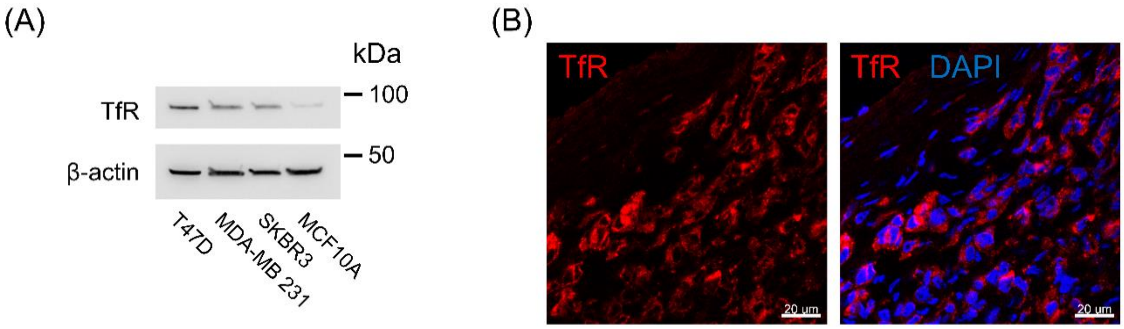

Supplementary Figure 3. Overexpression of transferrin receptor (TfR) in breast cancer cells and tissues. **(A)** Representative western blot of whole cell lysates of breast cancer cell lines vs non-cancerous MCF10A cell line probed with anti-TfR and anti-β-actin, as a loading control. **(B)** Representative confocal images of frozen T47D and MDA-MB-231 tumor xenograft sections immunostained with anti-TfR (red) and counterstained with DAPI (blue). Scale bar = 20 μm.

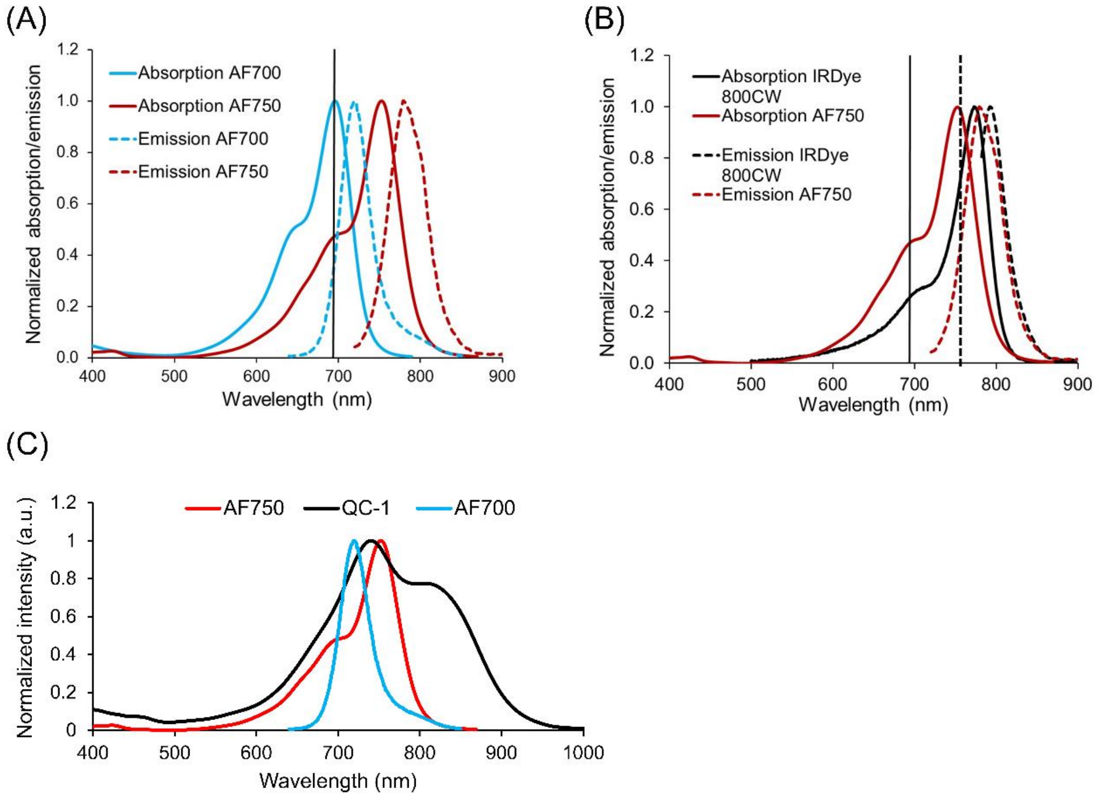

Supplementary Figure 4. (A) Absorption and emission spectra of FRET pair AF700/750. Vertical line indicates the laser excitation line 695 nm. **(B)** Absorption and emission spectra of AF750 and IRDye 800CW. Solid vertical line indicates 695 nm excitation (FRET MFLI imaging) and dotted vertical line indicates 750 nm excitation line (2-DG imaging). **(C)** Spectra overlap of donor AF700 emission spectrum and absorption spectra of acceptor AF750 or dark quencher acceptor QC-1.

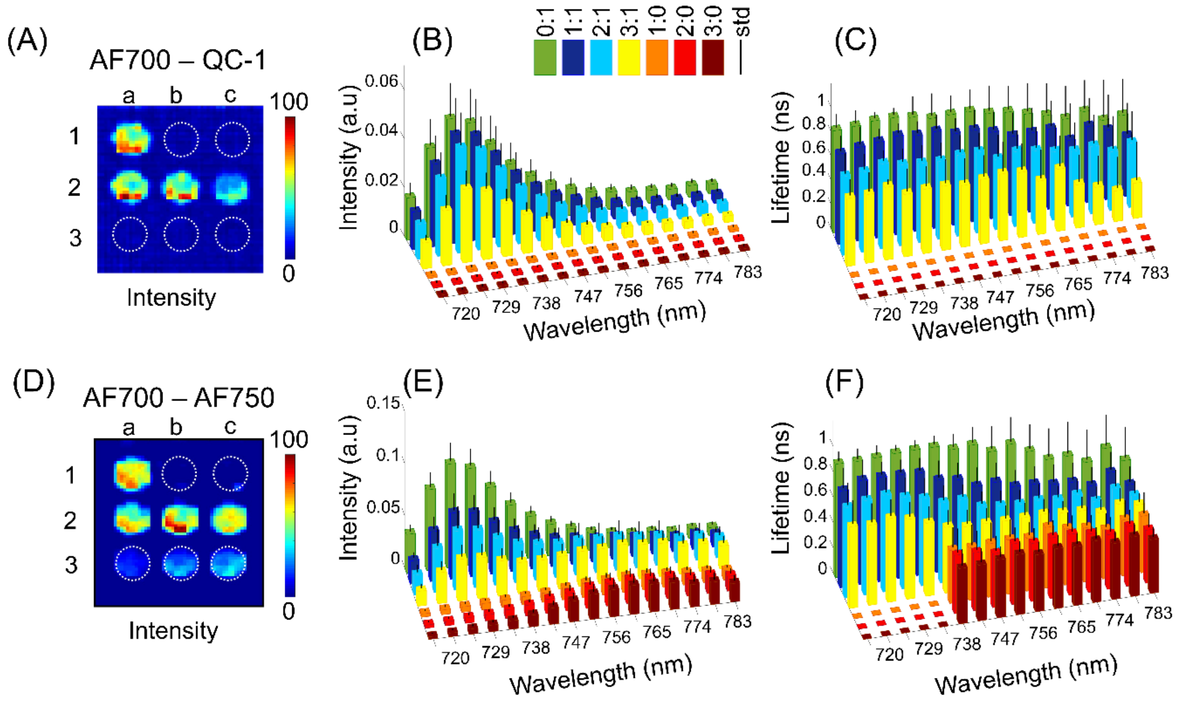

Supplementary Figure 5. Hyperspectral characterization of QC-1 as a FRET acceptor. **(A)** Reconstructed spectrally resolved continuous wave fluorescence intensity spatial map of AF700 donor and QC-1 acceptor at the 725 nm wavelength. Spectrally resolved mean intensity **(B)** and lifetime **(C)** distributions for each well per wavelength and A:D ratio for AF700/QC-1 FRET donor/acceptor sample. **(D)** Reconstructed spectrally resolved continuous wave (CW) intensity spatial map of AF700 donor and AF750 acceptor at the 725 nm wavelength. **(E)** Mean intensity and **(F)** lifetime per wavelength and A:D ratio for AF700/AF750 FRET donor/acceptor sample. For both **(A)** and **(D)**, the layout of the A:D ratios on the plate is as follows: a1-0:1, b1-0:0, c1-0:0, a2-1:1, b2-2:1, c2-3:1, a3-1:0, b3-2:0 and c3-3:0. Error bars represent standard deviation.

Supplementary Figure 6A

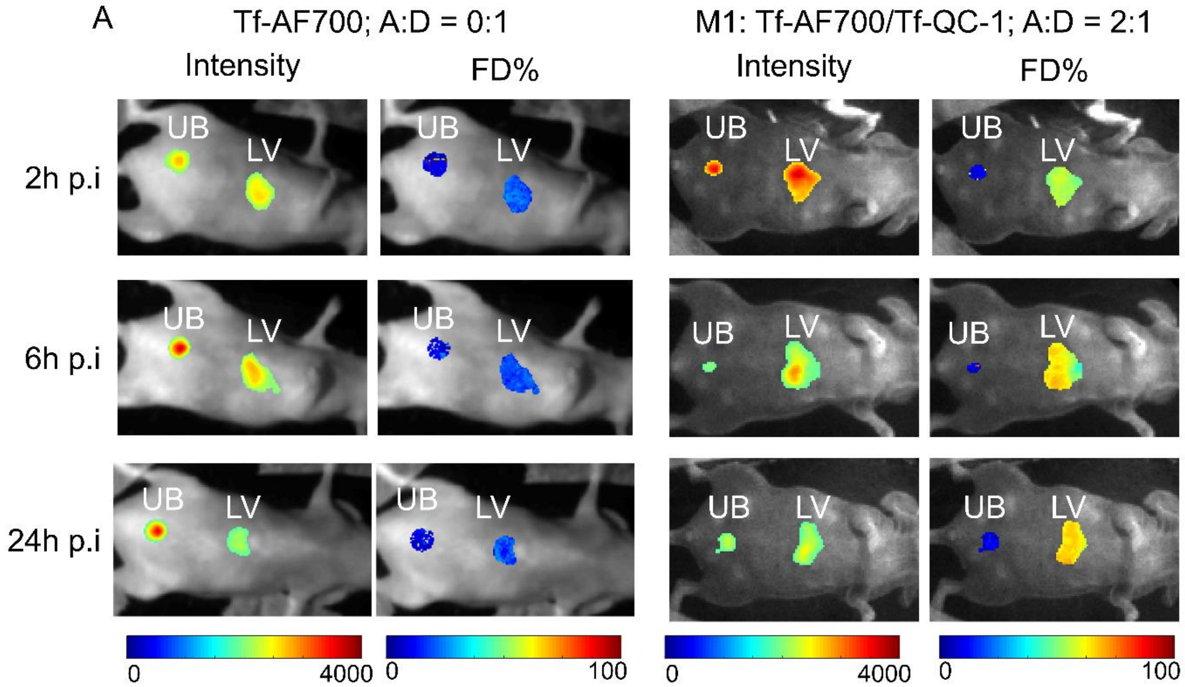

Supplementary Figure 6B

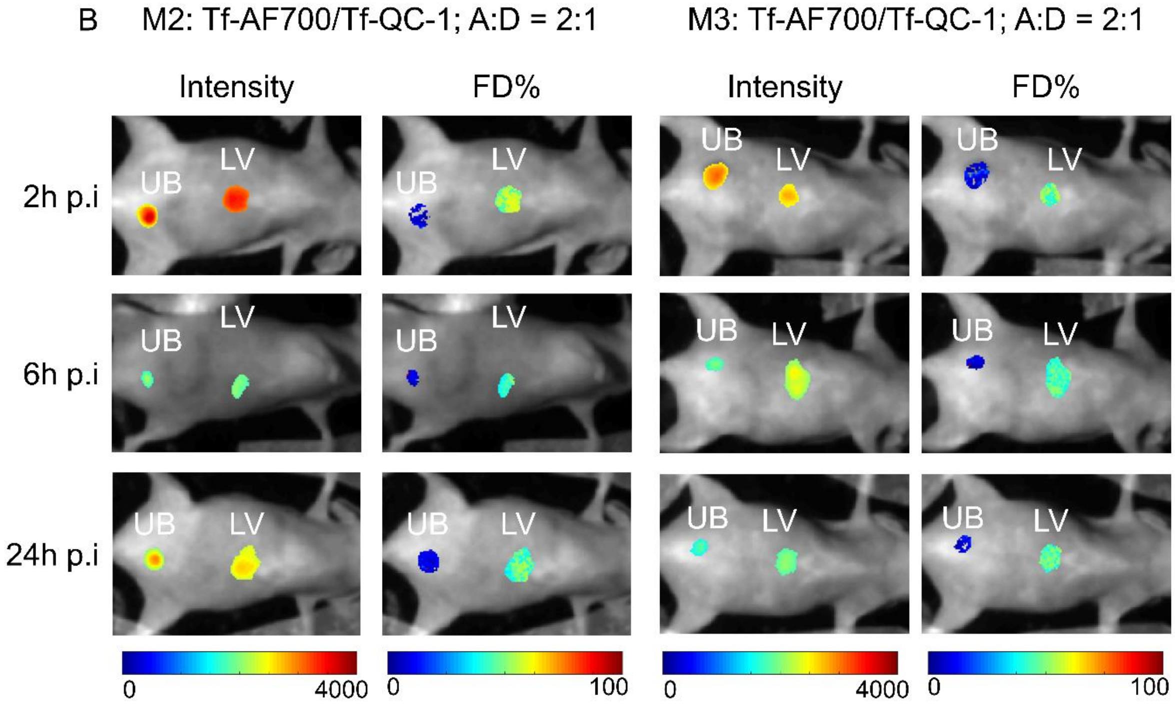

Supplementary Figure 6C

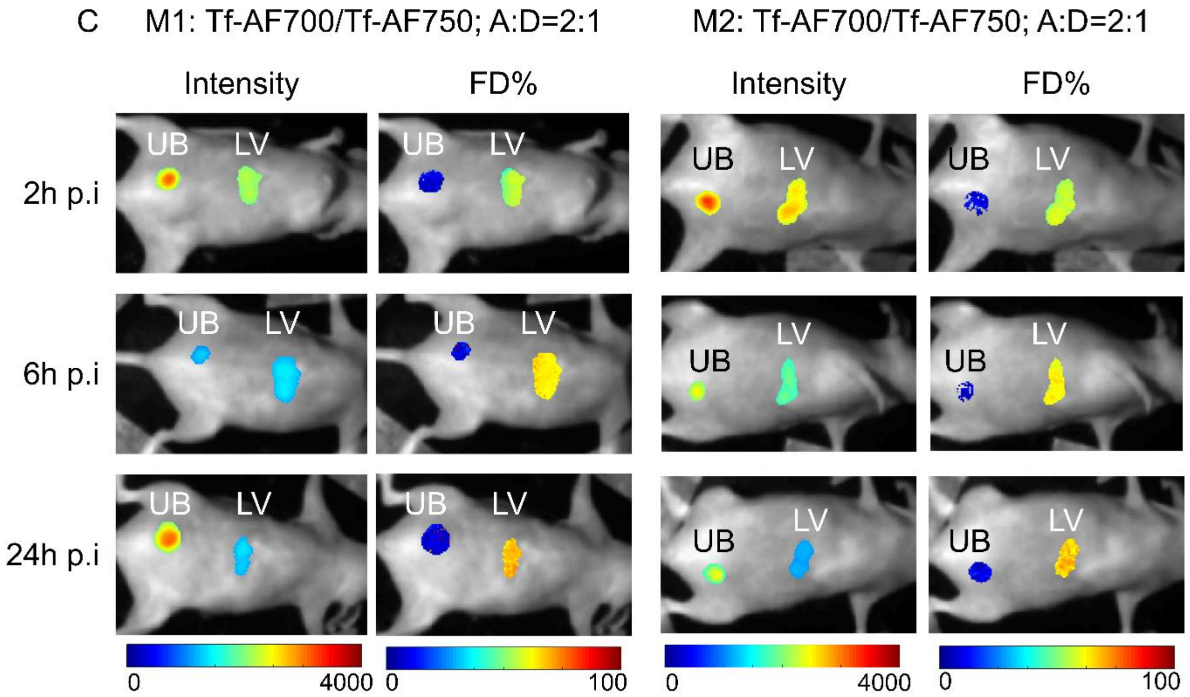

Supplementary Figure 6D

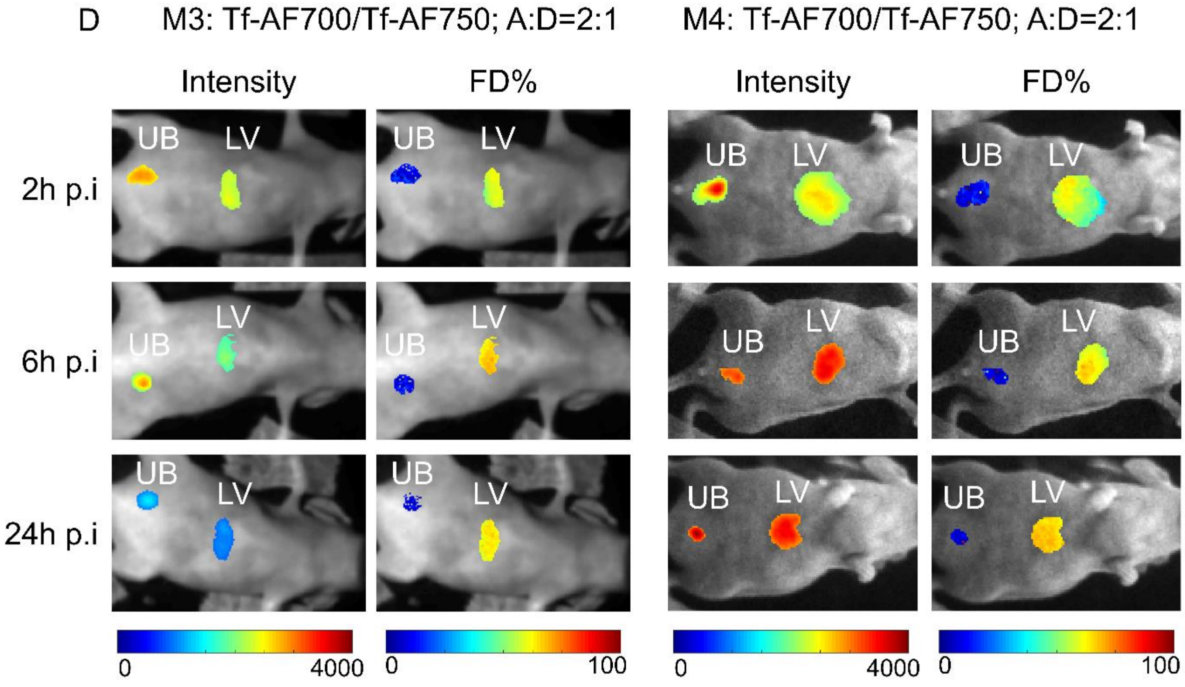

Supplementary Figure 6. Whole-body FRET MFLI *in vivo* imaging using dark quencher acceptor QC-1. Athymic nude mice were injected with either Tf-AF700/Tf-AF750 (M1-3; Panel A-B) or Tf-AF700/Tf-QC-1 (M1-4; Panel C-D) at A:D ratio of 2:1 and subjected to MFLI-FRET imaging at 2h, 6h and 24h p.i. One mouse was injected with Tf-AF700 (donor only control; Panel A). Panels show Tf donor fluorescence intensity maximum (total Tf, including soluble and bound Tf) and FD% levels (bound Tf) of live, intact mice using the MFLI-FRET imager. Within each mouse image, ROIs show liver and bladder fluorescence and FD% levels. These mice were used in the analysis shown in **Figure 5**.

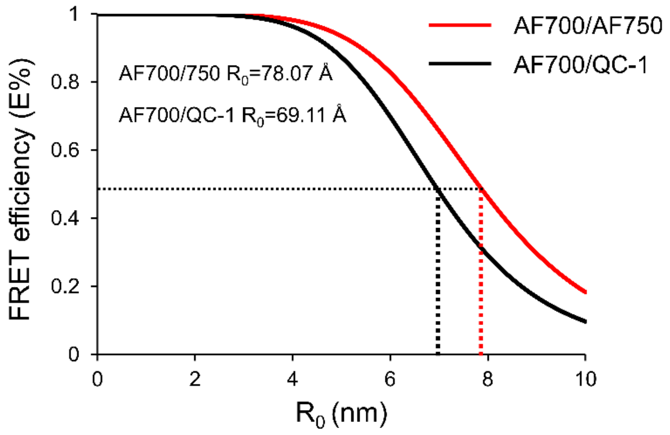

Supplementary Figure 7. FRET efficiency plot in relation to intrinsic intermolecular distance of AF700/750 and AF700/QC-1. Dashed lines indicate 50% FRET efficiency for AF700/AF750 at R_0 = 7.8 nm vs ~30% for AF700/QC-1 (R_0 = 6.9 nm) at the same distance. We have calculated R_0 for these NIR FRET pairs using standard calculations as described previously in [14,17,66,67].

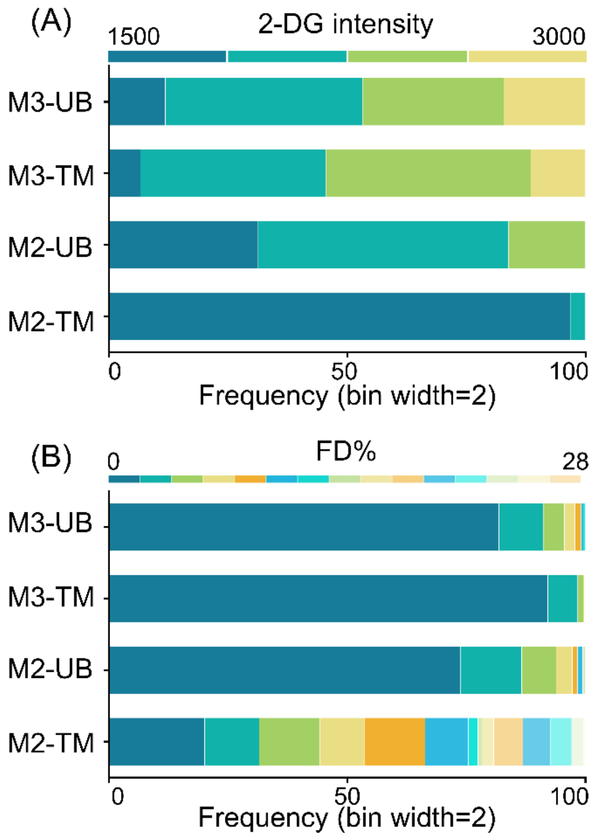

Figure S8. Distribution of 2-DG fluorescence intensity (**A**) and FD% signal (**B**) per ROI pixel in tumors and bladders from M2 and M3 mice. (**A**) Stacked frequency bar chart of 2-DG fluorescence intensity, bin width = 500 a.u. (**B**) Stacked frequency bar chart of Tf FD%, bin width = 2. Analysis of data from **Figure 7**.

Tumors	M1	M3	M2
Size (mm^3)	32	455.5	98.6
A:D ratios	0:1	2:1	2:1
TfR staining	++	++	++
FRET signal	-	+/-	++
Tf staining	+	+	++
2-DG signal	++	+++	+
GLUT1 staining	+++	++	+

Supplementary Figure 9. Relationship between tumor size and TfR, Tf and GLUT1 immunostainings and 2-DG and FRET MFLI imaging for mice imaged in **Figure 6-7**.

Supplementary Tables

**Supplementary Table 1**. Statistical analysis of FD% and 2-DG values between tumor and bladder (Figure 1)							
2-DG values	N total (pixels)	Mean	Standard Deviation	FD% values	N total (pixels)	Mean	Standard Deviation
Tumor	282	1434.451	738.386	Tumor	152	14.7343	3.9474
Bladder	310	959.535	229.936	Bladder	434	6.3733	3.7075
Two-sample t-test (Welch Correction)	p-value = 6.06169E-22 ($p < 0.05$; significant)			Two-sample t-test (Welch Correction)	p-value = 4.10768E-63 ($p < 0.05$; significant)		

**Supplementary Table 2.** Statistical analysis of AF700/QC1 vs. AF700/AF750 FRET pairs at increasing A:D ratios (Figure 2)			
Repeated measures Anova	p-value = 0.129404 ($p > 0.05$; not significant)	F = 3.631218	c 7.708647

**Supplementary Table 3.** Statistical analysis of TfR-Tf-AF700 vs. TfR-Tf-QC-1 colocalization, as indicated by Pearson's coefficient (Figure 3)					
	N	Mean Pearson's coefficient	Standard deviation	SEM	Median
TfR and Tf-AF700	5	0.6276	0.02188	0.00978	0.6168
TfR and Tf-QC-1	5	0.6626	0.02146	0.0096	0.6592
Two tailed t-test	p-value = 0.0888 ($p > 0.05$; not significant)				

**Supplementary Table 4.** Statistical analysis of Tf-AF700/Tf-QC1 vs. Tf-AF700/Tf-AF750 FRET pairs at increasing A:D ratios ($p < 0.05$) using repeated measures Anova (Figure 3C-F)			
Repeated measures Anova	p-value= 0.023491 ($p < 0.05$; significant)	F = 12.70571	$F\ crit$ = 7.708647

**Supplementary Table 5.** Statistical analysis for data presented in Figure 6.							
Fig. 6 Tumors	N total (pixels)	Mean	Standard Deviation	Tumors	N total (pixels)	Mean	Standard Deviation
M1 2-DG	45	1952.777	39.3474	M1 FD%	42	4.3811	4.1007
M2 2-DG	112	1491.607	172.325	M2 FD%	110	9.2705	4.1421
Two-sample t-test (Welch Correction): p-value = 8.17432E-56; $p < 0.05$; significant				Two-sample t-test (Welch Correction): p-value = 6.28815E-9; $p < 0.05$; significant			

Fig.6 M1	N total (pixels)	Mean	Standard Deviation	M2	N total (pixels)	Mean	Standard Deviation
tumor 2-DG	45	1952.777	39.3474	tumor 2-DG	112	1491.607	172.325
bladder 2-DG	309	2498.1974	382.2519	bladder 2-DG	569	1715.214	399.013
Two-sample t-test (Welch Correction): p-value = 3.83703E-76; $p < 0.05$; significant				Two-sample t-test (Welch Correction): p-value = 1.23526E-19; $p < 0.05$; significant			

Fig.6 M1	N total (pixels)	Mean	Standard Deviation	M2	N total (pixels)	Mean	Standard Deviation
tumor FD%	42	4.3811	4.1007	tumor FD%	110	9.2705	4.1421
bladder FD%	253	1.2391	1.9735	bladder FD%	502	3.1789	2.9625
Two-sample t-test (Welch Correction) p-value = 1.45209E-5; $p < 0.05$; significant				Two-sample t-test (Welch Correction) p-value = 1.39464E-29; $p < 0.05$; significant			

Supplementary Table 6. Statistical analysis for data presented in Figure 7.

Fig. 7 D-E Tumors	N total (pixels)	Mean	Standard Deviation	Tumors	N total (pixels)	Mean	Standard Deviation
M3 2-DG	655	2308.858	354.465	M3 FD%	431	0.221	0.989
M2 2-DG	250	1526.564	126.486	M2 FD%	244	7.936	7.293
Two-sample t-test (Welch Correction) p-value = 1.061x10-255; p < 0.05; significant				Two-sample t-test (Welch Correction) p-value = 1.418x10-41; p < 0.05; significant			

Fig.7 D-E M3	N total (pixels)	Mean	Standard Deviation	M2	N total (pixels)	Mean	Standard Deviation
tumor FD%	431	0.22131	0.98966	tumor FD%	244	7.9365	7.2933
bladder FD%	226	0.7448	1.85754	bladder FD%	179	1.0718	2.1455
Two-sample t-test (Welch Correction) p-value = 9.68959E-5; p < 0.05; significant				Two-sample t-test (Welch Correction) p-value = 3.18747E-34; p < 0.05; significant			

Fig.7 D-E M3	N total	Mean	Standard Deviation	M2	N total	Mean	Standard Deviation
tumor 2-DG	655	2308.858	354.465	tumor 2-DG	250	1526.564	126.487
bladder 2-DG	297	2261.431	413.639	bladder 2-DG	211	1931.057	253.967
Two-sample t-test (Welch Correction) p-value = 0.08761; p < 0.05; significant				Two-sample t-test (Welch Correction) p-value = 9.80633E-61; p < 0.05; significant			

**Supplementary Table 7.** Statistical analysis for data presented in Figure 7F (normalized to livers)							
Fig. 7 F-G Tumors	N total (pixels)	Mean	Standard Deviation	Tumors	N total (pixels)	Mean	Standard Deviation
M3 2-DG	655	1.0877	0.02171	M3 FD%	431	0.0049	0.2814
M2 2-DG	250	0.79718	0.06605	M2 FD%	244	0.3062	0.0217
Two-sample t-test (Welch Correction) p-value = 2.4311E-116; $p < 0.05$; significant				Two-sample t-test (Welch Correction) p-value = 2.65984E-42; $p < 0.05$; significant			
